# Unimodal vs. multimodal deep learning for non-invasive MGMT promoter methylation prediction in glioblastoma: A systematic evaluation on the *BraTS* 2021 dataset

**DOI:** 10.1371/journal.pone.0351405

**Published:** 2026-06-12

**Authors:** Freddy Oulia, Philippe Charton, Muhammad Kabir, Fabrice Gardebien, Cédric Damour, Frederic Cadet

**Affiliations:** 1 BIGR, Inserm, U1134, University Paris City, Paris, France; 2 Faculty of Sciences and Technology, DSIMB, BIGR, Inserm, U1134, University of Reunion, Saint-Denis, France; 3 Department of Computer Science, University of Management and Technology, Lahore, Pakistan; 4 Faculty of Sciences and Technology, ENERGYLab, University of Reunion, Saint-Denis, France; 5 PEACCEL, AI for Biologics, Paris, France; Goethe University Hospital Frankfurt, GERMANY

## Abstract

Glioblastoma multiforme (GBM) is the most aggressive primary brain tumor in adults, with a median survival of 14.6 months under standard radiotherapy and temozolomide (TMZ) chemotherapy. The methylation status of the O⁶-methylguanine-DNA methyltransferase (MGMT) promoter is a critical biomarker predicting TMZ response; however, its determination currently requires invasive tissue sampling. Non-invasive prediction of MGMT promoter methylation from multiparametric MRI (mpMRI) through deep learning represents a compelling alternative, yet its clinical feasibility remains unresolved. Using the BraTS 2021 dataset (582 patients, four MRI sequences: FLAIR, T1w, T1wCE, T2w), we conducted a systematic comparative study of unimodal and multimodal deep learning approaches based on VGG-16, exploring 1,380 experimental configurations (unimodal: 192; multimodal: 1,188) across three imaging planes, eight slice counts, and three multimodal fusion strategies (early, intermediate, and late fusion). In the unimodal setting, the best model trained on T2w coronal images (32 slices, no transfer learning) achieved an accuracy of 0.6458 and an AUC of 0.6422 on the validation set, but dropped to 0.5586 and 0.5533 on the independent test set, revealing substantial overfitting attributable to limited dataset size. Strikingly, multimodal fusion consistently failed to outperform the best unimodal model, with all three fusion strategies plateauing at ~0.64 accuracy and ~0.64 AUC on validation data. Transfer learning improved generalization across train/test distributions at the cost of peak performance. These findings suggest, for the tested framework in this study, that MGMT methylation status prediction from mpMRI remains fundamentally constrained by dataset heterogeneity and size, irrespective of modality combination strategy, and that T2w coronal acquisitions could be more interesting in future data collection efforts.

## Introduction

Gliomas account for approximately 30% of all brain tumors and 70–80% of malignant brain tumors, representing a heterogeneous group of neoplasms originating in glial cells of the central nervous system [1]. Among them, glioblastoma multiforme (GBM), classified as an isocitrate dehydrogenase wild-type diffuse astrocytic tumor in the 2021 WHO Classification of Tumors of the Central Nervous System [2], stands as the most aggressive and lethal primary brain tumor in adults [3]. GBM is characterized by rapid cellular proliferation, diffuse brain infiltration, and profound resistance to conventional therapies, collectively resulting in a dismal prognosis. Despite multimodal treatment combining surgical resection, radiotherapy (RT), and temozolomide (TMZ) chemotherapy — the current standard of care since the landmark Stupp et al. trial [4] — median survival remains limited to approximately 14.6 months [4]. The complex and heterogeneous genomic landscape of GBM [5], further formalized in the 2021 WHO classification [2], reflects the difficulty in developing effective targeted therapies and underscores the urgent need for reliable molecular biomarkers to guide personalized treatment decisions [6].

Among the molecular determinants of GBM prognosis, the methylation status of the O⁶-methylguanine-DNA methyltransferase (MGMT) promoter occupies a central position. In tumor cells, the MGMT protein repairs alkylation damage to DNA induced by TMZ, thereby conferring chemoresistance [7]. When the MGMT promoter is epigenetically silenced by methylation, this repair mechanism is impaired, rendering tumor cells susceptible to TMZ-induced apoptosis. Hegi et al. [8] demonstrated that patients harboring a methylated MGMT promoter exhibit a markedly improved prognosis, with a median survival of 21.7 months under RT combined with TMZ, compared to 15.3 months with RT alone. The MGMT promoter methylation status has since been established as a key predictive biomarker in clinical trials, guiding decisions on TMZ administration and avoiding exposing patients to ineffective, toxic, and costly treatments [9]. However, its determination currently relies on bisulfite sequencing or pyrosequencing of surgically resected tumor tissue — a procedure that is invasive, time-consuming, and subject to sampling bias due to intratumoral heterogeneity [10,11].

The possibility of inferring MGMT promoter methylation status non-invasively from preoperative magnetic resonance imaging (MRI) — a concept referred to as “virtual biopsy” or radiogenomics [12] — has attracted considerable research interest over the past decade. Multiparametric MRI (mpMRI), which simultaneously acquires complementary tissue contrast information through sequences such as Fluid-Attenuated Inversion Recovery (FLAIR), T1-weighted (T1w), T1-weighted with Contrast Enhancement (T1wCE), and T2-weighted (T2w) imaging, provides a rich, non-invasive characterization of tumor microenvironment, vascularity, and edema [13]. The BraTS 2021 challenge, organized jointly by the Radiological Society of North America (RSNA), the American Society of Neuroradiology (ASNR), and the Medical Image Computing and Computer Assisted Interventions (MICCAI) Society, provided the largest publicly available standardized mpMRI dataset for this task, encompassing 585 patients with all four MRI sequences [14–16].

Deep learning (DL) approaches, and convolutional neural networks (CNNs) in particular, have emerged as the dominant paradigm for MGMT status prediction from mpMRI. The winner of the BraTS 2021 radiogenomic classification challenge achieved an AUC of 0.622 on the private leaderboard using a 3D-ResNet10 trained exclusively on T1wCE images. Subsequent studies have explored a broader range of architectures — including 2D and 3D CNNs, Vision Transformers (ViT) [17], and Swin Transformers [18] — yet performance on the BraTS dataset has consistently plateaued around AUC 0.62–0.65, regardless of architectural complexity [19–21]. In contrast, models trained on smaller but more clinically homogeneous datasets such as The Cancer Imaging Archive (TCIA) [22] or The Cancer Genome Atlas (TCGA) [23] have reported considerably higher performance (AUC up to 0.81) [20], raising serious questions about the real-world generalizability of such models and the suitability of the BraTS 2021 dataset for this classification task [24,25]. Recent systematic reviews and critical evaluations have confirmed the absence of scientific consensus on whether MGMT promoter methylation status can be reliably inferred from MRI alone using current DL methods [19,26].

A key dimension that has received insufficient systematic attention in the literature is the comparative impact of modality selection — unimodal versus multimodal — and of acquisition parameters such as imaging plane and slice count on predictive performance. Most existing studies have either used all four MRI sequences by default, without evaluating the marginal contribution of each modality, or have reported results on a single modality without rigorous multimodal comparison. Furthermore, the three main strategies for multimodal fusion in deep learning — early fusion, intermediate fusion, and late fusion — have rarely been evaluated jointly and under controlled experimental conditions on the same dataset. It therefore remains unclear whether combining multiple MRI sequences systematically yields predictive gains, and if so, which fusion strategy and which modality combination prove most effective.

In this study, we address these open questions through a comprehensive and systematic experimental investigation on the BraTS 2021 dataset. Using a VGG-16 backbone — a CNN architecture with established performance in medical imaging tasks [27,28] — we trained and evaluated over 1,300 model configurations across two main experimental axes: (i) a unimodal approach, exploring all four MRI sequences across three imaging planes (axial, coronal, sagittal) and eight slice counts, with and without transfer learning initialization; and (ii) a multimodal approach, combining 2, 3, or 4 MRI sequences through early, intermediate, and late fusion strategies, under the same parametric grid. Our primary aim is not to propose a new architecture, but to systematically characterize which input features — modality, plane, slice count, and fusion strategy — yield the best-performing and most generalizable models, within the tested 2D VGG-16 framework, for MGMT promoter methylation prediction, thereby offering preliminary insights that could be more efficient for future data collection and model development in this clinically critical task.

## Methodology and methods

### Dataset description

The dataset provided by the BraTS challenge [14] is composed of a training and a testing set. The test set is strictly used to evaluate competitor’s model performance and, with labels not available, this set cannot be used in this study. The usable dataset is initially composed of 585 instances, but 3 patients must be removed due to some discrepancies (patients: 00109, 00123, 00709) – 306 methylated patients and 276 unmethylated patients making an effective dataset of 582 patients. In this study, the remaining data is split into train and test sets with a ratio 8:2 with respect to the initial distribution with a random seed for reproducibility.

For each patient, 4 different mpMRI are available and each MRI sequence display and highlight different information:

- **FLAIR:** it is designed to suppress the cerebrospinal fluid (CSF) signals in the image to enhance the visibility of lesions near the ventricles and in white matter. This is particularly useful for detecting strokes, infections, or tumors.
- **T1w:** this sequence highlights anatomical detail by emphasizing fat-rich tissues while making water and CSF appear darker providing clear differentiation of gray and white matter. It is valuable for anatomical studies, assessing brain structure or detecting hemorrhages.
- **T1wCE:** this sequence is obtained by administrating a gadolinium-based contrast agent before a T1 imaging. It will highlight tumors, infections, inflammation or vascular abnormalities.
- **T2w:** it emphasizes water content by making fluid appear bright and fat appear darker. Detection of edema, inflammation or any lesions with high water content is improved with this sequence.

**Fig 1:**
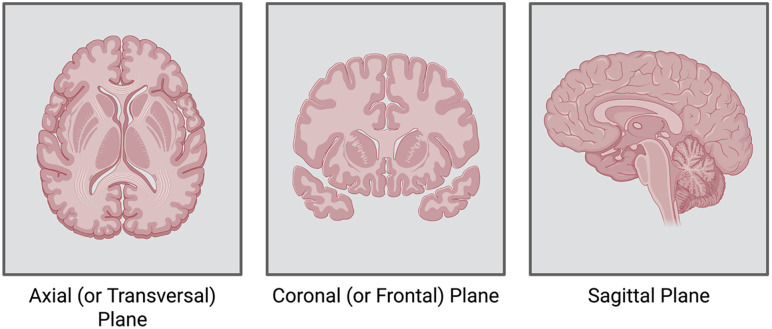
Different orientation planes of brain images. In the axial plane, the brain is visualized either from the top of the skull to the bottom or the other way around. The coronal plane displays scans from the face to the back of the head (or the opposite direction). Finally, the sagittal plane shows scans from one ear to the other.

The DICOM format stores 3D scans as a list of 2D images. The 3D scan is cut into 2D slices, and each slice is saved as a file. Table 1 below provides different information about the number of files in each MRI sequence such as the total number of files or the average number of files per patient. There are 3 planes in which we can visualize these successive 2D images along an axis: axial, coronal, and sagittal. Fig 1 shows these 3 different planes.

**Table 1:** Summary of the total number of files, maximum, minimum, and average number of files per patient for each MRI modality.

	FLAIR	T1w	T1wCE	T2w
**Total number of files**	74,010	77,128	96,368	99,774
**Maximum number of files for a single patient**	514	400	400	472
**Minimum number of files for a single patient**	15	19	19	19
**Average number of files per patient**	127	132	165	171

When visualizing 3D scans with 2D images, 3 axes can be used: the axial plane, the coronal plane, and the sagittal plane. Fig 1 shows these 3 different planes.

### Pre-processing

All mpMRI scans were acquired from various scanners from multiple institutions and under different clinical protocols. Therefore, there are irregularities between the different scans. Before distributing the dataset, the challenge provider pre-processed the scans: they are co-registered to the same anatomical template, interpolated to the same resolution (1 mm^3^) and skull stripped.

In this study, further pre-processing steps are done prior to feeding these scans into CNN models. When picking 2D slices from the 3D images, the image orientation is not consistent between different patient and MRI sequences. The first step is to uniformize the orientation for each scan by explicitly selecting a 2D plane to use. Then, scans are centered around the brain, and a cropping process is used to remove parts of the scans that do not contain any information. Each scan is then resized to 224*224, standard image size for 2D CNN models. When manipulating MRI images for ML/DL models, each scan must be normalized with either min-max normalization or z-score standardization.

Min-max normalization:


$$\begin{array}{c} x^{\prime }=\frac{x-x_{min}}{x_{max}-x_{min}} \end{array}$$ (1)

with $x^{\prime }$ the normalized value, x the original value, and $x_{min}$, $x_{max}$ the minimal and maximal value of the set of data.

Standardization:


$$\begin{array}{c} z=\frac{x\;-\;\mu }{\sigma } \end{array}$$ (2)

with z the standardized value, x the original value, μ the mean of the dataset, and σ the standard deviation.

**Fig 2:**
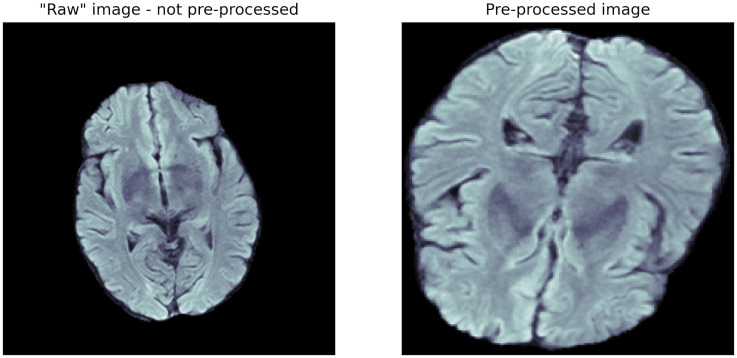
Comparison between non-pre-processed and pre-processed scans. The left image is a brain scan under the FLAIR MRI sequence, and we did not apply any pre-processing on this image. The right image is the same after the pre-processing steps: re-orientation, cropping to center the image, resizing, and standardization.

For this study, the standardization is used with its parameters computed for each scan. In the final pre-processing step, a defined number of slices is extracted symmetrically around the central slice of the scan. When a single slice is selected, only the central one is retained; when multiple slices are chosen, the selection extends outward from the center in both directions. Fig 2 shows a comparison of the same brain image before and after the pre-processing steps applied in this work.

### Baseline

**Fig 3:**
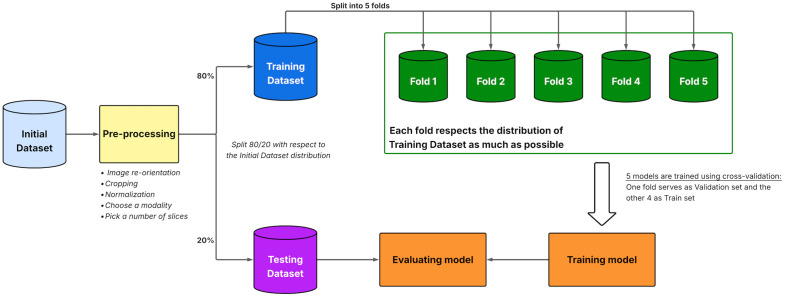
Cross-validation workflow to train and evaluate models. Before training models, the chosen MRI sequence is picked with the number of 2D slices. Then, the dataset is split into a train set and a test set. A 5-folds cross-validation is done with the train set and the 5 trained models are evaluated on the same test set previously set aside.

The baseline approach used in this study, where we train a 2D CNN model using cross-validation is described in Fig 3. From the initial dataset, a single MRI sequence (FLAIR, T1w, T1wCE, or T2w) is selected alongside the number of 2D slices used to train the model. The channel dimension in the first convolutional layers is adapted to match the number of 2D slices. A random set of 20% of patients (test set) is held out and never used during training or any model selection decision, thus avoiding any risk of data leakage. The test set is strictly used to evaluate the generalization capacity of the model. A 5-fold cross-validation is exclusively applied on the remaining 80% training partition. Models are trained using CrossEntropyLoss as loss function with a batch-size of 64 and an EarlyStopping function is set up to prevent overfitting. The model’s parameters are optimized with Adam. The metrics used to evaluate the performance of the model are accuracy and AUC score.

Transfer Learning (TL), where model parameters are extracted from models already trained on a large dataset, can help to speed up the training and potentially yield better results. To determine if TL brings better results, the 5-folds cross-validation is run twice, once with the already trained parameters and once without. When using TL, parameters in the convolutional layers are frozen and not trained except when replacing the first convolutional layers to match the channel dimension with the number of slices.

In this work, we use the VGG-16 model that has already proved its relevance in bioinformatics. Ghosh et al. [27] used this model to perform brain tumor segmentation and reached 0.997-pixel accuracy outperforming other common CNN models. In the study of Xu et al. [28], constructed a VGG16-based CNN lung screening model to effectively identify lung tumors and the model demonstrated an AUC of 0.963 highlighting its high precision. The primary aim of this study is to conduct a controlled, exhaustive combinatorial evaluation of input configurations (sequence, plane, …) without a variance caused by a different architecture. Using a 2D backbone model such as VGG-16 instead of a 3D model provides a more computationally tractable framework.

### Multimodal approach

With each out of the 4 MRI sequences highlighting different elements in brain scan, using a single modality for predicting MGMT promoter methylation status is limiting. A multimodal approach, combining simultaneously 2 or more MRI sequences, is a promising solution to have more robust results. There are 3 different ways to implement a multimodal approach with DL:

- Early fusion is the simplest method to incorporate multiple modalities in a single model. S1 Fig in the Supplementary file describes this method. Each modality is processed in the model through the input layer, and they are directly merged into a single instance. In this study, MRI sequences are stacked one on top of the other (e.g., with T1w and T2w, the tensor is composed of every slice from the T1w sequence and then followed by every slice of the T2w sequence). The whole tensor is then forwarded in the convolutional and classification layers to make a prediction using the modalities.
- The second method used in this study is the intermediate fusion where each sequence is processed separately in the same convolutional layers to extract features specific to each modality. Then, features can either be summed, averaged, or concatenated into one tensor before the classification layers. The latter solution is used in this study. S2 Fig in the Supplementary file shows this workflow.
- Finally, the last multimodal approach used in this study is the late fusion method. For each sequence, features are extracted, and a decision (or prediction) is made separately. Each modality is processed in the same model and using all decisions, a final prediction is made. There are multiple ways to implement the final decision such as majority voting or weight voting. In this study, we decided to concatenate each decision into a tensor and forwarded it in a neuron layer to make the final decision. S3 Fig in the Supplementary file describes this method.

## Results

### Unimodal results

With the approach described in the subsection Baseline in section Methodology & Methods, the model is trained after selecting the MRI sequence to use alongside the number of 2D slices for each scan (1, 3, 8, 16, 24, 32, 40 or 48) and the orientation plane and every possibility is explored. In each case, the model is trained and evaluated once with model parameter initialization using TL and a second time without TL. The results are the averages obtained during cross-validation with 5 folds.

**Fig 4:**
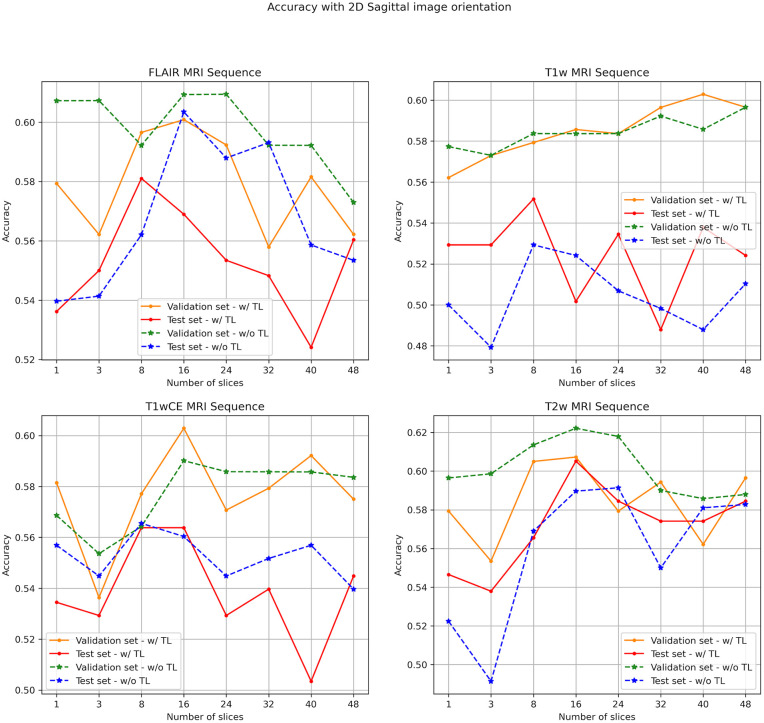
Accuracy results on each modality based on the number of slices used with 2D scans from the sagittal plane. In each figure there are results with a model trained with Transfer Learning and a model trained without TL. Best results on validation sets are with the T2w modality and 16 slices: 0.6221 without TL and 0.6073 with TL. On the test set, the same model reaches an accuracy of 0.5897 without TL and 0.6052 with TL.

Fig 4 shows accuracy results, in validation and test sets, for each modality when using 2D scans in the sagittal plane. For each modality, there are results with different numbers of slices used and there is a set of results with a model trained with and without TL. Supplementary S4 and S5 Figs show more precise results for images on the sagittal plane with a heatmap of results with a model trained with Transfer Learning and a heatmap of results without Transfer Learning. Best accuracy results on validation sets are exhibited with T2w MRI sequence with a mean accuracy of 0.6221 (with a standard deviation of 0.0449) when using 16 slices and model trained without TL. When using TL, accuracy peaks at 0.6073 (± 0.0447) when using the same images. With this MRI sequence and number of slices, the accuracy on the test set reaches 0.5897 (± 0.0069) without TL and 0.6052 (± 0.0342) with TL. In each modality, performance on the validation set tends to be close between using TL and not using TL and with, interestingly, slightly better results with models trained without TL. However, results on test sets are less consistent and patterns noticed on results on validation sets are less visible on test sets.

**Fig 5:**
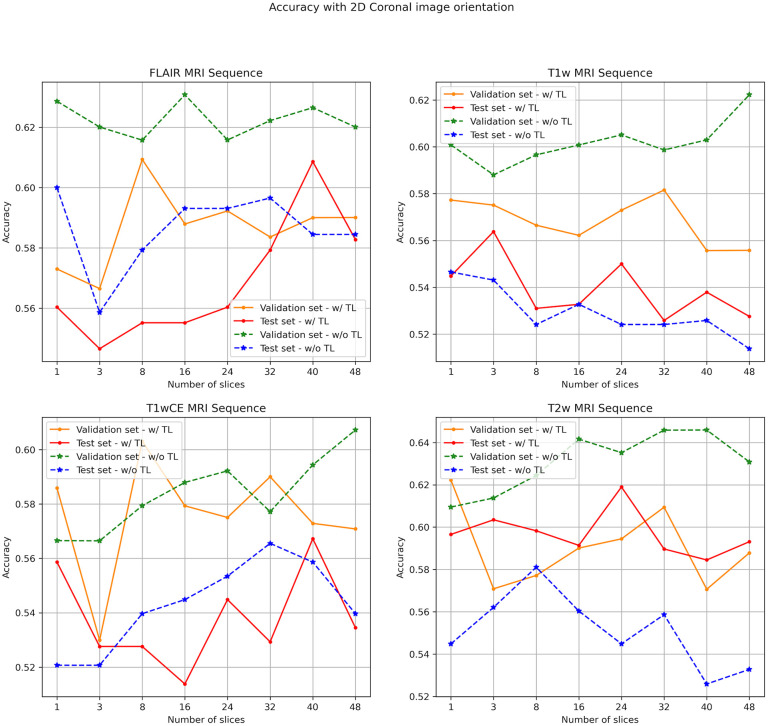
Accuracy curves result on each modality based on the number of slices used with 2D scans from the coronal plane. In each figure there are results with a model trained with TL and a model trained without TL. Best results on validation sets are with the T2w modality and 40 slices: 0.6459 without TL and 0.6222 with TL and using only 1 slice. On the test set, these same models have an accuracy of 0.5259 and 0.5966 respectively.

Similarly to the previous figure, Fig 5 displays accuracy results, in validation and test sets, for each modality when using 2D scans but in the coronal plane this time. More precise results using the coronal plane are presented in Supplementary file with S6 Fig for models trained with TL and S7 Fig for models trained without TL. With this plane, the best overall result on the validation set is obtained by using T2w images with 32 slices and the accuracy is 0.6458 ± 0.0348 (without TL). However, on the test set, accuracy is down at 0.5259 (± 0.0184). When using Transfer Learning to initialize a model’s parameters, the best model is obtained by using T2w images with only 1 slice. The performance is a bit more consistent with 0.6222 ± 0.0351 on the validation set and 0.5966 ± 0.0492 on the test set.

Finally, with scans in the axial plane, the same trends can be identified on the results, i.e., performance on the validation set is slightly better with models trained without TL and the difference of performance between validation set and test set is also slightly greater. Figures with accuracy curves and the heatmaps are in the Supplementary file (S8–S10 Figs). With these scans too, the best performances on the validation set are observed with T2w images. The difference in performance between validation set and test set is also significant.

**Fig 6:**
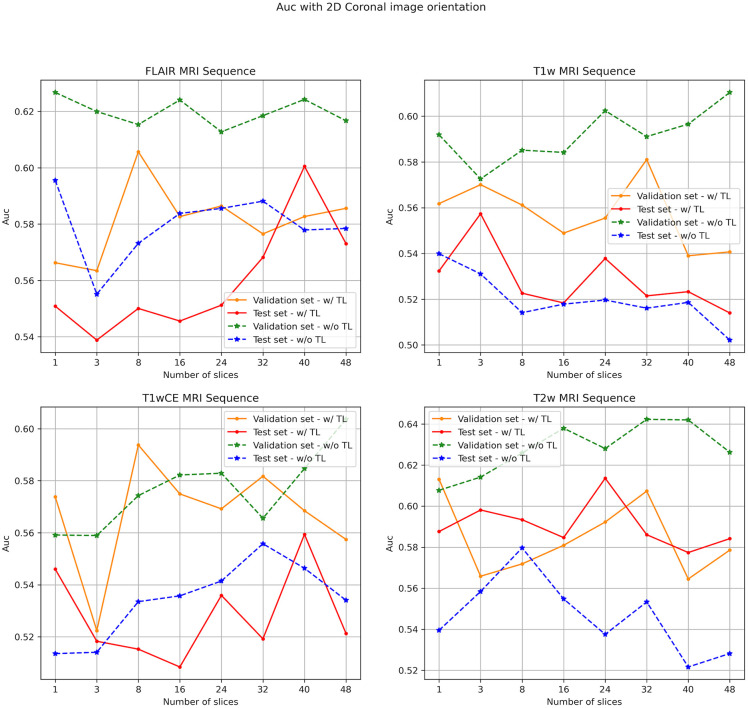
AUC curves result on each modality based on the number of slices used with 2D scans from the coronal plane. In each figure there are results with a model trained with TL and a model trained without TL. Best results on validation sets are with the T2w modality and 32 slices for a model trained without TL (0.6422) and with a single slice for a model trained with TL (0.6131). On the test set, these same models have an AUC of 0.5533 and 0.5876 respectively.

Concerning the AUC metric, the same observations made on the accuracy results can be done. The best results on the validation set are generally obtained with T2w images and models trained without TL. Similarly, the difference of performance between the validation set and test set is significant. Fig 6 shows AUC results with scans on the coronal plane and more complete results are presented in the Supplementary file (S11 Fig for results with TL and S12 Fig for results without TL). The model with the best AUC on the validation is trained using T2w images with 32 images and reaches 0.6422 ± 0.0388. However, on the test set, the model has an AUC of 0.5533 ± 0.0182 with the same condition. The same remarks are made for the other type of image orientation but with slightly lower results.

Table 2 allows a contextualization of our best results obtained with the unimodal approach against the literature. The test set used by the competition winner is not available to us. The study by Robinet et al. uses a private dataset, and Saeed et al. does not have a test set since they use cross-validation. Our model, which achieves its best performance on the validation data in terms of both accuracy and AUC, was trained with images from the T2w sequence in a coronal plane with 32 image slices, without initialization with TL. This model achieves an AUC of 0.6422 (± 0.0388) and an accuracy of 0.6458 (± 0.0348) on the validation data, but the performance on the test data is lower with 0.5533 (± 0.0182) and 0.5586 (± 0.0184). This difference in performance between the validation and test data can be explained by the small size of the dataset (582 patients). It is reasonable to suggest that an increase in the database would, at a minimum, bring the performance of the validation and test data closer together.

**Table 2:** Best results obtained with a unimodal approach. The results presented in this table are means (± standard deviation) taken from all folds during cross-validation. *Note*: * indicates that the results are difficult to compare with ours since we do not have access to their data (private data or retained by the competition organizer). *NA*: Information Not Available from various studies.

Model	MRI Sequence	Slices	Plane	Transfer Learning	Validation Accuracy	Validation AUC	Test Accuracy	Test AUC
**VGG-16** *This study*	T2w	32	Coronal	No	0.64 ± 0.03	0.64 ± 0.04	0.56 ± 0.02	0.55 ± 0.023
**VGG-16** *This study*	T2w	1	Coronal	Yes	0.62 ± 0.03	0.61 ± 0.04	0.59 ± 0.05	0.59 ± 0.05
**3D-ResNet10** *Winner*	T1wCE	32	*NA*	No	*NA*	*NA*	*NA*	0.62*
**3D-ResNet10** *Robinet et al*.[19*]*	FLAIR	*NA*	*NA*	Yes	0.62*	0.65*	*NA*	*NA*
**ResNet-34** *Saeed et al*.[20*]*	FLAIR	*NA*	*NA*	*NA*	*NA*	0.61	*NA*	*NA*

### Multimodal results

Based on the four MRI sequences and combining 2, 3, and 4 sequences simultaneously, there are a total of 11 possible combinations. For each combination, six models are trained with each time a different number of slices (1, 3, 8, 16, 24, and 32), and this is repeated twice to initialize model parameters once with Transfer Learning and the second time without TL. These steps are then repeated for each of the orientation planes for the scans (coronal, axial, and sagittal). Given these parameters, the number of models to train is 396 (11 * 6 * 2 * 3 = 396). In this work, the multimodal approach is explored through 3 methods: early fusion, intermediate fusion, and late fusion. Therefore, the total number of models to train and evaluate is 1,188 (396 * 3 = 1,188).

Using the early fusion method, the model having the best accuracy and AUC performance on the validation set uses a combination of T1w, T1wCE and T2w images on the coronal plane with 16 slices for each modality. The model is trained without TL and reaches an accuracy of 0.6417 (with a standard deviation of 0.0316) and an AUC of 0.6392 (± 0.0318) on the validation set. As the results from the unimodal approach, the performance on the test set is noticeably lower with an accuracy of 0.5759 ± 0.0246 and an AUC of 0.5718 ± 0.0246.

From the intermediate fusion, it is the combination of T1w and T2w sequences on the coronal plane with 16 slices that produces the best accuracy (0.6393 ± 0.0381) and AUC score (0.6364 ± 0.0419) on the validation set when the model is not trained with TL. However, the performance on the test set only reaches 0.5569 (± 0.0159) and 0.5521 (± 0.0177) respectively.

Similarly with the late fusion method, it is the same model that has the best accuracy and AUC score on the validation set. The model is trained with a combination of FLAIR and T2w images on the coronal plane with 16 images and without TL. The accuracy on the validation set reaches 0.6416 ± 0.0149 but only 0.5724 ± 0.0291 on the test set. Regarding the AUC score, the model obtains a score of 0.6391 ± 0.0162 on the validation set and 0.5651 ± 0.0308 on the test set.

Table 3 presents some of our best models obtained with a multimodal approach. For each type of fusion, we present the accuracy and AUC performances of two models (the best with TL initialization and the best without TL initialization). We find that for each of our models; the AUC and accuracy scores are very close to each other on the same dataset (difference starts from the third decimal place). In the case of selecting the best-performing models, there does not seem to be one type of fusion that performs better than the others or one type of fusion that performs worse. The best models have equivalent performance. In addition, we note that the best-performing models frequently use coronal images. The combination of two sequences, in most cases, is sufficient to generate the best models, and the T2w sequence is the most frequently involved. T1w and T1wCE sequences also appear frequently in the best models.

**Table 3:** Best results obtained with a multimodal approach. The results presented in this table are means (± standard deviation) taken from all folds during cross-validation. *Note*: * indicates that the results are difficult to compare with ours since we do not have access to this data (private data or retained by the competition organizer). *NA*: Information Not Available from various studies.

Model	MRI Sequence	Slices	Plane	TL	Type fusion	Val Accuracy	Val AUC	Test Accuracy	Test AUC
**VGG-16** *This study*	T1w, T1wCE, T2w	16	Coronal	No	Early	0.64 ± 0.03	0.64 ± 0.03	0.57 ± 0.02	0.57 ± 0.02
**VGG-16** *This study*	T1w, T2w	8	Coronal	Yes	Early	0.59 ± 0.03	0.59 ± 0.03	0,61 ± 0.02	0,61 ± 0.02
**VGG-16** *This study*	T1w, T2w	16	Coronal	No	Intermediate	0.64 ± 0.04	0.64 ± 0.04	0.56 ± 0.02	0.55 ± 0.02
**VGG-16** *This study*	T1wCe, T2w	8	Sagittal	Yes	Intermediate	0.55 ± 0.01	0.55 ± 0.02	0.61 ± 0.03	0.61 ± 0.04
**VGG-16** *This study*	FLAIR, T2w	16	Coronal	No	Late	0.64 ± 0.01	0.64 ± 0.02	0.57 ± 0.03	0.56 ± 0.03
**VGG-16** *This study*	T1w, T2w	1	Axial	Yes	Late	0.59 ± 0.05	0.59 ± 0.04	0.63 ± 0.04	0.62 ± 0.04
**3D-ResNet10** *Robinet et al.* [19]	FLAIR, T1wCE	*NA*	*NA*	No	Early	0.62*	0.64*	*NA*	*NA*
**ResNet-34** *Saeed et al.* [20]	All	*NA*	*NA*	*NA*	*NA*	*NA*	0.61	*NA*	*NA*
**EfficientNet-b1** *Saeed et al.* [20]	All	*NA*	*NA*	*NA*	*NA*	*NA*	0.63	*NA*	*NA*

Results in Table 3 are accompanied by multimodal results found in the state-of-the-art. In the study of Robinet et al., they use a private test set not accessible for us. Saeed et al. uses cross-validation to train their model; therefore, they do not have any results on a test set.

## Discussion

### Performance plateau and the intrinsic difficulty of MGMT prediction from the BraTS 2021 dataset

The most salient finding of this study is that neither unimodal nor multimodal deep learning approaches succeeded in exceeding an AUC of approximately 0.64 on the validation set using the BraTS 2021 dataset, with test-set performance consistently lower (~0.55). Based on our results, the hypothesis of a performance ceiling can be made, however it is unclear if it is primarily dataset-related or methodology-related. A safe assumption is to put responsibility on both aspects. This performance ceiling is fully consistent with the broader literature on this specific dataset. The competition winner of the BraTS 2021 radiogenomic challenge achieved an AUC of only 0.622 on the private leaderboard, and subsequent large-scale validation studies have provided an even more sobering assessment. Kim et al. [29] conducted 420 training and validation experiments across multiple CNN architectures, MRI sequences, and datasets, reporting that over 80% of models showed no statistically significant difference from chance-level performance (50%) in terms of test accuracy when validated on an independent external cohort (SNUH, n = 400). The first-place BraTS challenge solution itself degraded to an AUC of 0.562 on this external test set. Robinet et al. [19] corroborated these findings, concluding that deep learning algorithms developed to date on this dataset are not suitable for clinical application. These results tend to shift the responsibility of the performance ceiling to be primarily dataset related.

A plausible explanation for this hard ceiling lies in the nature of the binary labeling scheme used in the BraTS 2021 dataset, where MGMT status is assigned based on a pyrosequencing threshold of 10% methylation. As noted by Robinet et al. [19], cases with methylation percentages just below or just above this threshold are radiogenomically indistinguishable, yet are assigned opposite class labels, introducing irreducible label noise that any classification algorithm will struggle to overcome. This structural ambiguity in the ground truth — inherent to the dataset design rather than to any particular modeling choice — provides a principled explanation for the universal performance plateau observed across architectures and input configurations. Our results extend and reinforce this hypothesis by demonstrating that the ceiling is robust not only across model types but also across all three fusion strategies and across the full combinatorial space of slice count, imaging plane, and transfer learning initialization.

### The T2w coronal plane: a consistent but modest advantage

Within the tested 2D VGG-16 framework on the BraTS 2021 dataset, T2w coronal images were consistently associated with the best-performing configurations. This finding may reflect dataset-specific imaging properties and should not be generalized without validation on independent cohorts and architectures. The T2w sequence is sensitive to water content and tissue edema, which are hallmarks of peritumoral infiltration in GBM. The coronal plane, by providing a full cross-sectional view of the temporal lobes and peritumoral edema extent, may capture spatial patterns of tumor infiltration that are less visible in the axial plane, the most commonly used orientation in prior studies. This observation is partly convergent with findings from Yu et al. [30], who reported that peritumoral regions carry discriminative information for MGMT prediction, with their combined intratumoral/peritumoral model on the TCIA dataset reaching an AUC of 0.923. Beyond T2w, no other single modality produced decisively inferior results, suggesting that the discriminative information available across all four MRI sequences is approximately equivalent in this dataset — a finding that itself may reflect dataset-level limitations rather than true biological equivalence between sequences.

### Multimodal fusion does not improve predictive performance

A central and robust finding of this study is that combining multiple MRI sequences through any of the three fusion strategies — early, intermediate, or late — does not yield better-performing models than the best unimodal approach within the tested 2D VGG-16 framework. This result is both surprising and clinically important. The prevailing assumption in the field is that multiparametric MRI, by simultaneously encoding complementary tissue properties (edema, vascularity, necrosis, cellularity), should provide a richer feature space for radiogenomic classification than any single sequence alone. While this hypothesis has been validated in studies using curated, tumor-region-specific datasets — e.g., Yu et al. [30] on TCIA — it does not hold on the BraTS 2021 dataset under whole-image, slice-based approaches. The behavior observed in our model suggests that, at least in the present experimental setting, performance bottleneck appear to stem primarily from insufficient feature specificity and quality, particularly the absence of explicit tumor region localization, rather than from a lack of input modality diversity. This interpretation is supported by Saeed et al. [20], who similarly found that combining all four modalities did not consistently improve performance over single-sequence models on the BraTS dataset, and by Koska and Koska [31], who demonstrated that domain-knowledge-guided tumor mask integration could break through the performance ceiling that whole-image approaches systematically hit. In this study, the best-performing models obtained for each evaluated fusion strategy reached peak performance levels similar to those observed with the unimodal approach, despite the extensive set of experiments conducted using a common backbone architecture. This observation may indicate that the limited benefit of multimodality in this context is not primarily attributable to architectural design choices but could instead reflect underlying data-related constraints.

### Transfer learning: A trade-off between peak performance and generalization

Our results reveal a consistent and interpretable trade-off in the role of Transfer Learning. Models initialized without TL achieved slightly higher peak performance on the validation set, while TL-initialized models produced more stable and consistent results across validation and test sets. This pattern can be explained by the mismatch between the source domain of the pretrained VGG-16 weights (ImageNet, containing natural images) and the target domain (grayscale MRI slices). Without TL, models are free to learn representations entirely adapted to the MRI domain, potentially overfitting the validation set due to the small dataset size. With TL, the initialization with ImageNet features — while suboptimal for MRI textures — acts as a regularizer, dampening overfitting and improving the stability of generalization to the test set. This finding aligns with the broader literature on TL in medical imaging, where the benefit of pretrained weights is modulated by domain shift and dataset size. It also suggests that the observed validation-test gap is primarily a function of dataset size rather than architectural inadequacy: with 582 patients, even a 5-fold cross-validation leaves limited data for training, and the models’ ability to generalize remains structurally constrained.

### Clinical implications: Sensitivity-specificity trade-off, precision and F1 score

From a clinical standpoint, the sensitivity and specificity profiles of our models reveal an important asymmetry: most models exhibit relatively good sensitivity (60–72%), meaning that they tend to correctly identify methylated patients who would benefit from TMZ, but substantially lower specificity (45–65%), meaning that a significant proportion of unmethylated patients would be incorrectly predicted as methylated. Sensitivity is defined as:


$$\begin{array}{c} Sensitivity\;=\;TP\;/\;(TP\;+\;FN) \end{array}$$

where TP denotes true positives (biomarker methylated and correctly predicted) and FN false negatives (biomarker methylated but incorrectly predicted as unmethylated).

Specificity is defined as:


$$\begin{array}{c} Specificity\;=\;TN\;/\;(TN\;+\;FP) \end{array}$$

where TN denotes true negatives (biomarker unmethylated and correctly predicted) and FP false positives (biomarker unmethylated but incorrectly predicted as methylated). Table 4 below reports the sensitivity and specificity of the six best multimodal models (first six rows of Table 3) on validation and test sets.

**Table 4:** Sensitivity and specificity of our models in Table 3. The sensitivities and specificities presented in this table correspond to the models in the first 6 rows of Table 3.

Validation – Sensitivity	Validation – Specificity	Test – Sensitivity	Test – Specificity
0.6857	0.5927	0.6492	0.4945
0.6245	0.5701	0.7213	0.4909
0.5918	0.6519	0.6098	0.5018
0.5265	0.5796	0.6131	0.6145
0.6857	0.5923	0.7082	0.4218
0.5796	0.6068	0.7016	0.5491

The results in Table 4 are consistent with those observed for the unimodal models (Table 2). Sensitivity values range from 0.53 to 0.72 across configurations, while specificity values remain between 0.42 and 0.65, confirming the asymmetric error profile discussed above. In the clinical context of GBM, this asymmetry has distinct consequences: false positives expose unmethylated patients to TMZ-associated toxicity without therapeutic benefit, while false negatives deprive methylated patients who could benefit from the therapy. Neither error type is clinically acceptable at the rates observed in this study.

Precision and F1 score are another metrics to further the investigation about the clinical relevance of our models. Precision measures the proportion of correctly identified positive cases among all samples predicted as positive, thereby reflecting the model’s ability to limit false-positive predictions. A high precision indicates that positive predictions are highly reliable. The F1-score, defined as the harmonic mean of precision and sensitivity, provides a single summary metric that balances the trade-off between correctly identifying positive cases and avoiding missed detections. High F1 score implies that the model can both detect methylated cases effectively and maintain robustness against false alarms. The model trained with 32 slices of T2w coronal images without TL have a precision of 0.62 and a F1 score of 0.67 on the validation set and 0.57 and 0.61 respectively on the test set. For the model initialized with TL and trained with 1 slice of T2w coronal image, the precision is 0.62 and the F1 score is 0.68 on the validation set. On the test set, the metrics are 0.59 and 0.66. Table 5 shows the precision and F1 score for the best multimodal approach in the same manner of Table 4.

**Table 5:** Precision and F1 score of our models in Table 3. The precisions and F1 scores presented in this table correspond to the models in the first 6 rows of Table 3.

Validation – Precision	Validation – F1 score	Test – Precision	Test – F1 score
0.6538	0.6666	0.5885	0.6153
0.6277	0.6011	0.6157	0.6605
0.6631	0.6652	0.5707	0.6046
0.5942	0.5381	0.6439	0.6248
0.6594	0.6633	0.5779	0.6345
0.6187	0.5661	0.6346	0.6639

Taken together, our results confirm that the models presented here, consistent with the broader state of the art on the BraTS 2021 dataset, do not meet the clinical reliability threshold required for deployment in a treatment decision pipeline. This conclusion reinforces the assessment of Robinet et al. [19] that current DL algorithms trained on this dataset are not suitable for clinical application, and echoes the findings of Kim et al. [29] who concluded that MGMT methylation status in gliomas may not be predictable from preoperative MR images even using deep learning, at least not without substantially improved datasets.

### Study limitations

Several limitations of this study deserve explicit acknowledgment.

*Absence of tumor segmentation.* A major limitation of this study is the absence of tumor region segmentation, both in slice selection and at the model input level. The discriminative signal for MGMT methylation is likely concentrated in the tumor and peritumoral tissue, and whole-brain slice inputs introduce substantial irrelevant background signal that may dilute learned representations. Integration of explicit tumor segmentation—whether manual or automated (e.g., via U-Net-based approaches)—represents a primary avenue for extending this work, with the potential to enhance the signal-to-noise ratio and improve model performance.

*2D slice-based architecture.* The VGG-16 architecture, while well-validated in medical imaging classification tasks, is a 2D model applied to multi-slice stacks, which does not fully exploit the 3D spatial relationships inherent in volumetric MRI data. 3D architectures such as 3D-ResNet or 3D-EfficientNet may capture inter-slice structural patterns more effectively. It is noteworthy that the BraTS 2021 3D model winner exhibited a decrease in performance when evaluated on an independent dataset (Kim et al. [29]).

*Absence of interpretability analysis.* The lack of confidence calibration and explainability methods (e.g., Grad-CAM activation maps) limits our ability to determine whether models attend to tumor-relevant regions or to confounding imaging artifacts. Such analysis would be a necessary complement to any clinical translation attempt.

*Limited evaluation metrics.* The evaluation primarily relies on accuracy and AUC, without incorporating calibration or decision curve analyses. Although additional metrics (e.g., precision and F1-score) were reported for the best-performing models, the absence of calibration assessment limits insight into the reliability of predicted probabilities. This aspect should be addressed in future work to support more comprehensive model evaluation.

*Single split design.* The dataset was partitioned into training and test sets using a fixed random seed to ensure reproducibility. While this approach guarantees consistent splits across runs, it introduces a risk of seed-dependent variance, which represents a less robust alternative to methodologies such as nested cross-validation. However, the current pipeline already operates near its computational limits despite parallelization and runtime optimizations. Expanded computational capacity would be a prerequisite for moving toward a more reliable evaluation strategy.

*Single-dataset evaluation.* The study is confined to the BraTS 2021 dataset, and the generalizability of these findings to independently acquired clinical cohorts remains to be established. As demonstrated by Kim et al. [29], models trained and validated on BraTS may not transfer to institutional datasets, highlighting the importance of external validation as an essential step before any clinical claim.

*Single-framework evaluation.* The observations reported in this study are based on a single architectural framework (2D VGG-16), making it challenging to determine whether the observed effects are dataset-related or framework-dependent. While findings in prior work tends to suggest a stronger influence of dataset characteristics, this remains inconclusive in the present setting. Reproducing the pipeline across more recent and diverse architectures would therefore be necessary to assess the robustness and generality of these findings.

## Conclusion

This study presents a systematic investigation of deep learning-based prediction of MGMT promoter methylation status from mpMRI in GBM patients, addressing both unimodal and multimodal imaging strategies across a comprehensive set of architectural and acquisition configurations.

In the unimodal setting, the best-performing model exploited T2w images in the coronal plane without TL initialization, reaching 0.6458 accuracy and 0.6422 AUC on validation data. However, this performance degraded substantially on the independent test set (accuracy: 0.5586; AUC: 0.5533), a gap attributable to the inherent limitation of the BraTS 2021 dataset size (n = 582). Across all MRI sequences, T2w in the coronal plane consistently produced the most competitive models, while no other single modality exhibited decisively inferior results.

The multimodal experiments (encompassing 1,188 trained models across early, intermediate, and late fusion strategies) led to a key negative result: combining two, three, or four MRI sequences does not improve predictive performance beyond the best unimodal configuration. All fusion strategies converged to similar performance ceilings (~0.64 accuracy, ~ 0.64 AUC on validation), with no statistically meaningful differentiation between fusion methods. Notably, two-sequence combinations proved sufficient to match the performance of four-modality models, with T2w remaining the most recurrently involved sequence across top-performing configurations.

Regarding Transfer Learning, our results demonstrate a consistent trade-off: random initialization yielded higher peak performance on the validation set, while TL produced more stable generalization between validation and test sets. This observation supports the hypothesis that the primary bottleneck is dataset size rather than model capacity or feature richness.

Taken together, these findings reveal a hard performance ceiling for MGMT methylation prediction from the BraTS 2021 dataset that cannot be overcome by multimodal fusion alone. The clinical deployment of such models would require, at minimum, substantially larger and more diverse cohorts. Based on our empirical findings, T2w coronal images appear to be more suited to develop a better model, with T1w and T1wCE as strong secondary modalities. Beyond data augmentation, future work should explore richer input representations, including radiomics features, genomic co-variates, or longitudinal imaging, which may provide complementary discriminative signal currently absent from MRI alone. The integration of explainability methods (e.g., Grad-CAM) could further help identify the anatomical regions most predictive of methylation status, offering both biological insight and a pathway toward clinically actionable models.

## Supporting information

S1 Fig Workflow to use early fusion in a multimodal approach. First, each modality is concatenated into a single instance. In this study, we stack modalities one on top of the others in a single tensor. While on this figure 3 modalities are used, in this study, 2, 3, or 4 modalities can be used with this method. The data is then forwarded in the convolutional layers to extract features and finally in the classification layers to obtain the prediction.
(TIFF)

S2 Fig Workflow for intermediate fusion. In this multimodal approach, data from each modality is forwarded in the convolutional layers to extract features separately. For each modality, the same features extraction layers are used. Sets of features are then merged into one tensor by summing, averaging, or by stacking them (solution used in this study). Finally, features are processed in the classification layers to make the prediction. Similarly to the previous figure, in this study, this method is used with 2, 3 and 4 modalities.
(TIFF)

S3 Fig Late fusion workflow. Each modality is completely processed separately: features are extracted and a decision is made for the single modality with the same model used each time. The final decision is made by taking the prediction of each modality into account. In this study, each decision is forwarded in a single layer of neurons (fine-tuned during training) to make the final prediction. In this work, late fusion is used with 2, 3, or 4 modalities.
(TIFF)

S4 Fig Heatmap of accuracy results when using 2D scans on the sagittal plane and when the model is trained with parameters initialized with Transfer Learning. Training is done with 5-folds cross validation and results are averaged (for validation set and test set).
(TIFF)

S5 Fig Heatmap of accuracy results when using 2D scans on the sagittal plane and when the model is trained with parameters initialized without Transfer Learning. Training is done with 5-folds cross validation and results are averaged (for validation set and test set).
(TIFF)

S6 Fig Heatmap of accuracy results when using 2D scans on the coronal plane and when the model is trained with parameters initialized with Transfer Learning. Training is done with 5-folds cross validation and results are averaged (for validation set and test set).
(TIFF)

S7 Fig Heatmap of accuracy results when using 2D scans on the coronal plane and when the model is trained with parameters initialized without Transfer Learning. Training is done with 5-folds cross validation and results are averaged (for validation set and test set).
(TIFF)

S8 Fig Accuracy curves result on each modality based on the number of slices used with 2D scans from the axial plane. In each figure, there are results on validation set and test set with a model trained with Transfer Learning and a model trained without Transfer Learning.
(TIFF)

S9 Fig Heatmap of accuracy results when using 2D scans on the axial plane and when the model is trained with parameters initialized with Transfer Learning. Training is done with 5-folds cross validation and results are averaged (for validation set and test set).
(TIFF)

S10 Fig Heatmap of accuracy results when using 2D scans on the axial plane and when the model is trained with parameters initialized without Transfer Learning. Training is done with 5-folds cross validation and results are averaged (for validation set and test set).
(TIFF)

S11 Fig Heatmap with AUC results on validation sets and the test set with the model trained with Transfer Learning using 2D scans on the coronal plane. Training is done with 5-folds cross validation and results are averaged (for validation set and test set).
(TIFF)

S12 Fig Heatmap with AUC results on validation sets and the test set with the model trained without Transfer Learning using 2D scans on the coronal plane. Training is done with 5-folds cross validation and results are averaged (for validation set and test set).
(TIFF)
