# Supplementary figures and images for: Unimodal vs. multimodal deep learning for non-invasive MGMT promoter methylation prediction in glioblastoma: A systematic evaluation on the BraTS 2021 dataset
Source: PLoS One. 2026 Jun 12;21(6):e0351405. doi: 10.1371/journal.pone.0351405 (PMC13262868; doi:10.1371/journal.pone.0351405)

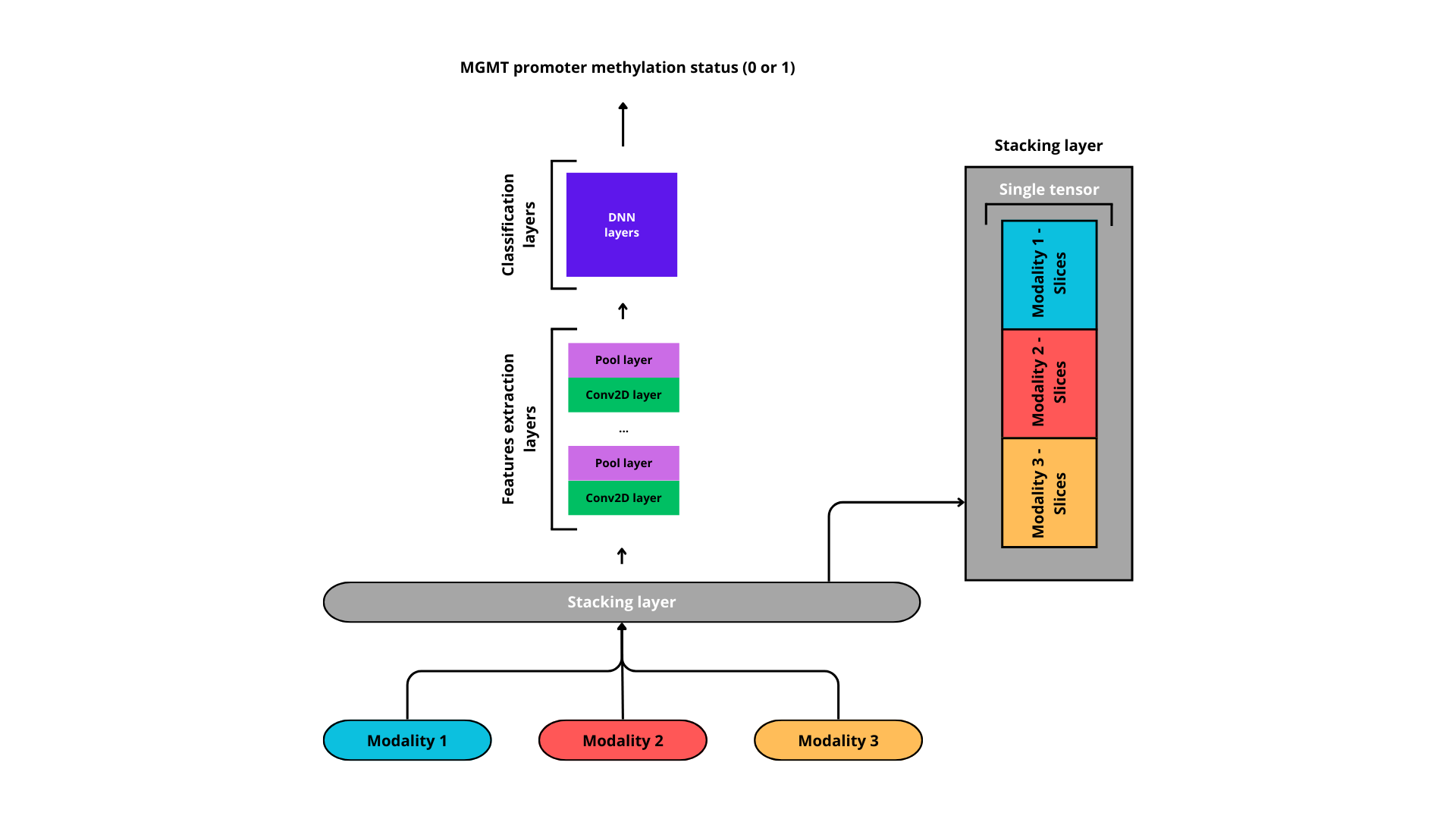

Supplement: S1 Fig — First, each modality is concatenated into a single instance. In this study, we stack modalities one on top of the others in a single tensor. While on this figure 3 modalities are used, in this study, 2, 3, or 4 modalities can be used with this method. The data is then forwarded in the convolutional layers to extract features and finally in the classification layers to obtain the prediction. (TIFF) [file pone.0351405.s001.tiff]

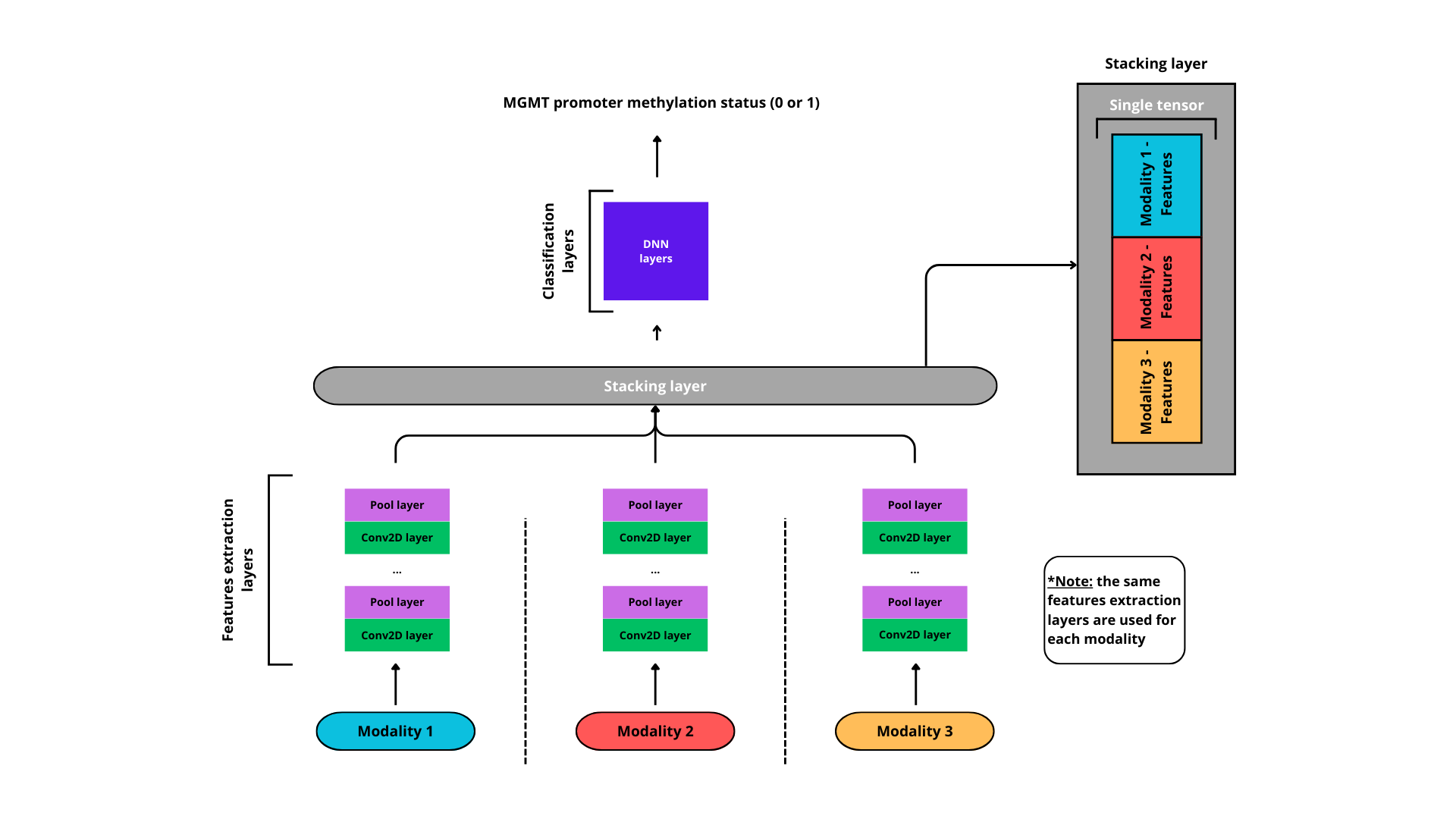

Supplement: S2 Fig — In this multimodal approach, data from each modality is forwarded in the convolutional layers to extract features separately. For each modality, the same features extraction layers are used. Sets of features are then merged into one tensor by summing, averaging, or by stacking them (solution used in this study). Finally, features are processed in the classification layers to make the prediction. Similarly to the previous figure, in this study, this method is used with 2, 3 and 4 modalities. (TIFF) [file pone.0351405.s002.tiff]

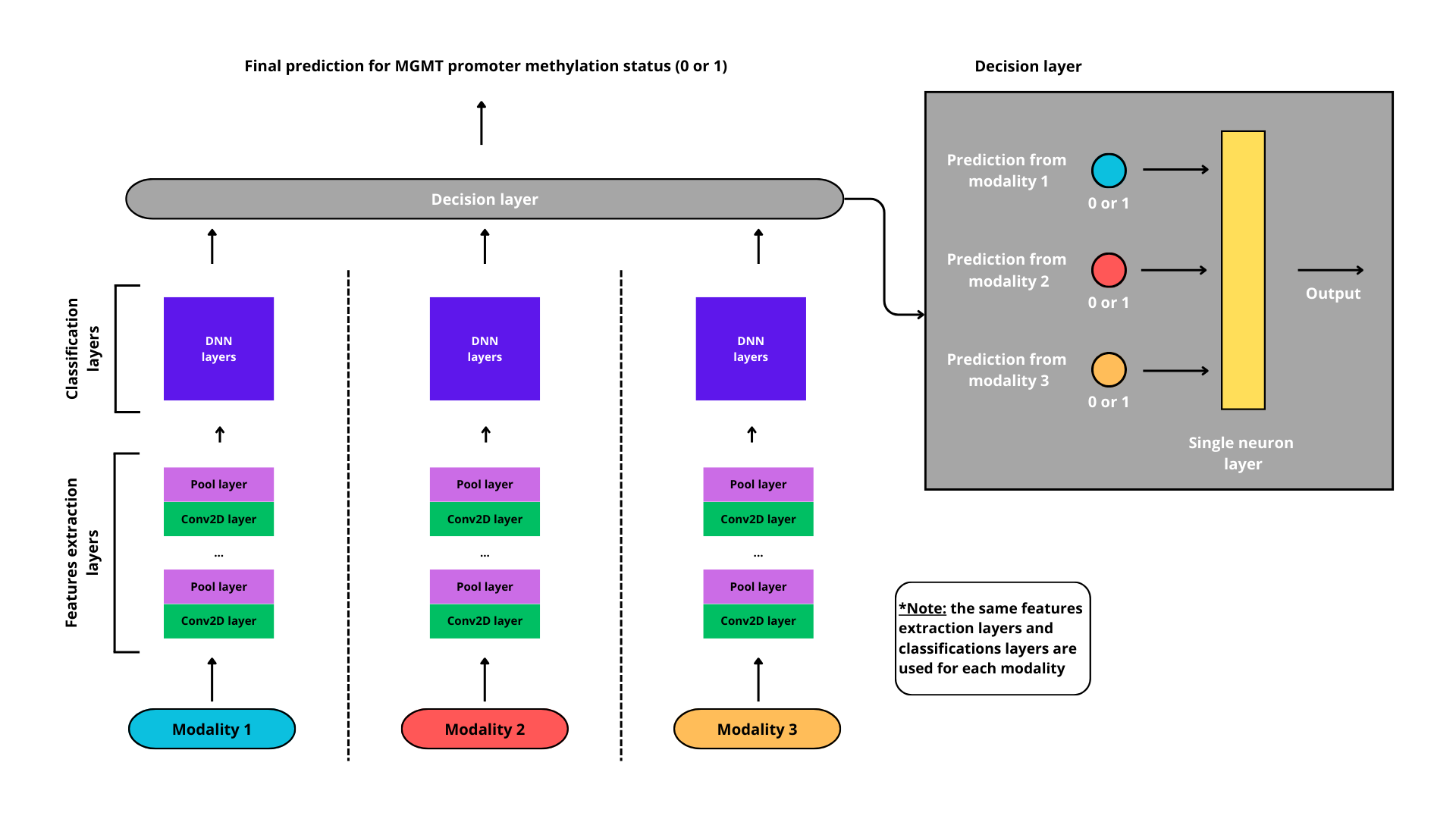

Supplement: S3 Fig — Each modality is completely processed separately: features are extracted and a decision is made for the single modality with the same model used each time. The final decision is made by taking the prediction of each modality into account. In this study, each decision is forwarded in a single layer of neurons (fine-tuned during training) to make the final prediction. In this work, late fusion is used with 2, 3, or 4 modalities. (TIFF) [file pone.0351405.s003.tiff]

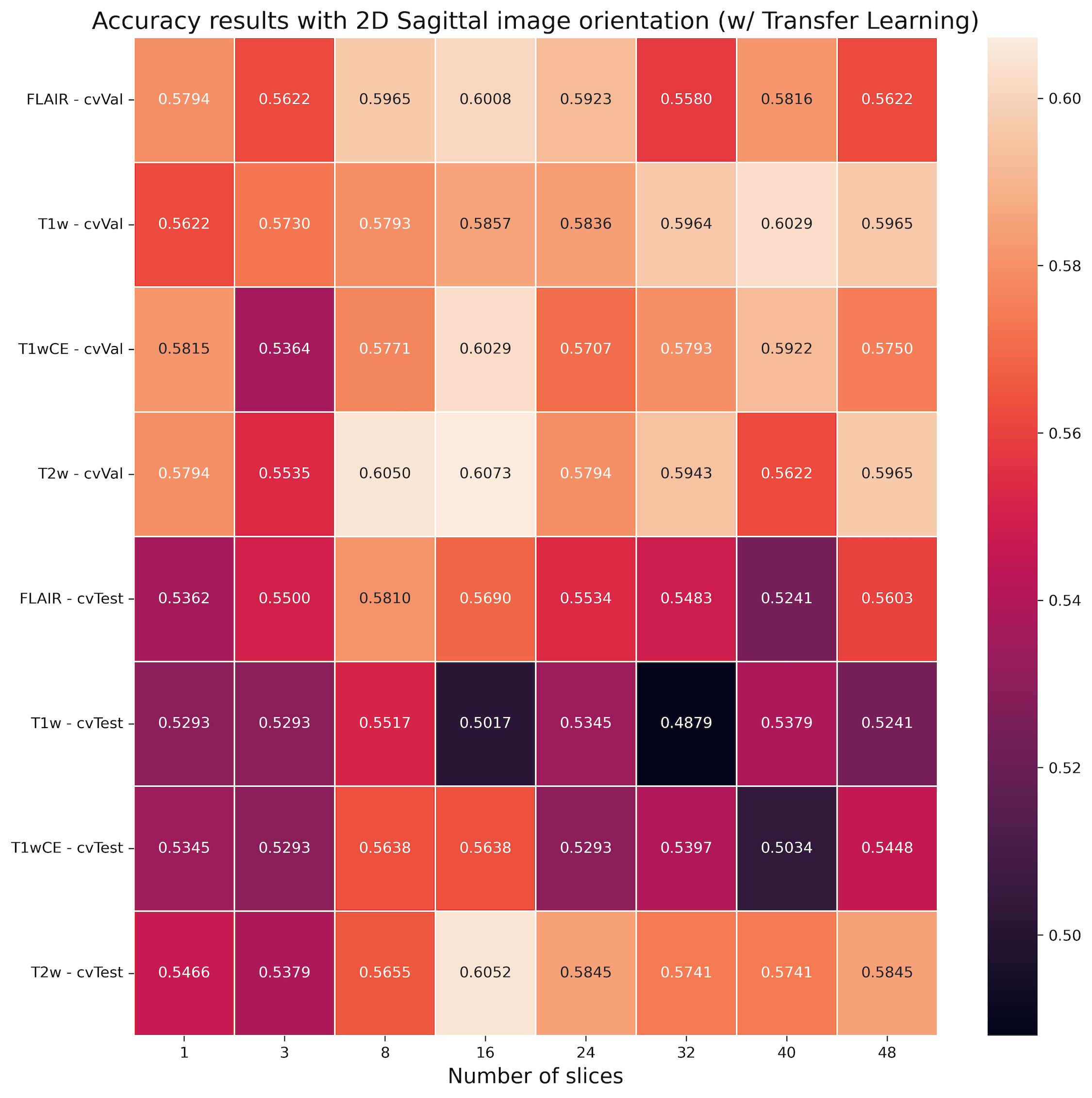

Supplement: S4 Fig — Training is done with 5-folds cross validation and results are averaged (for validation set and test set). (TIFF) [file pone.0351405.s004.tiff]

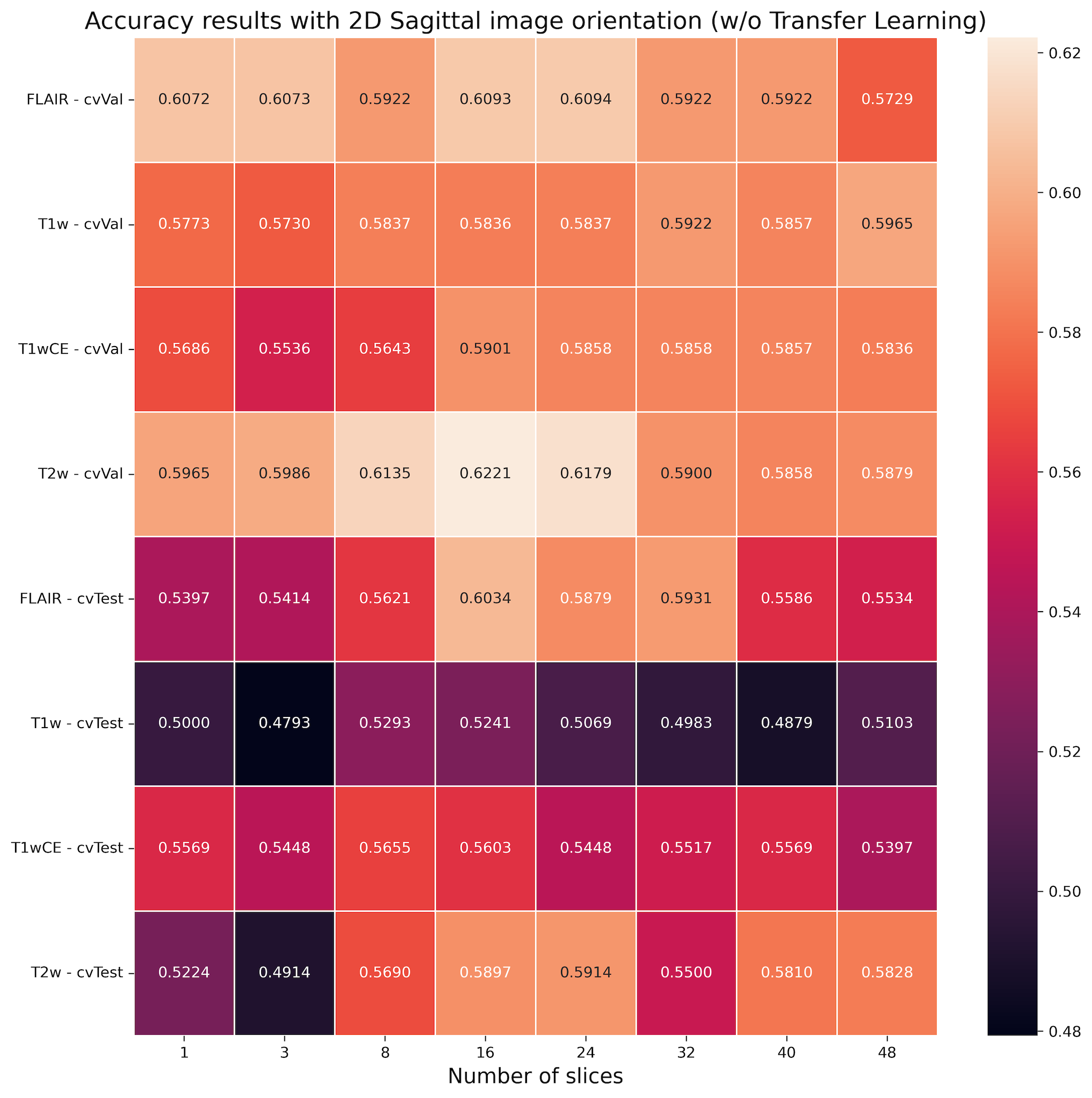

Supplement: S5 Fig — Training is done with 5-folds cross validation and results are averaged (for validation set and test set). (TIFF) [file pone.0351405.s005.tiff]

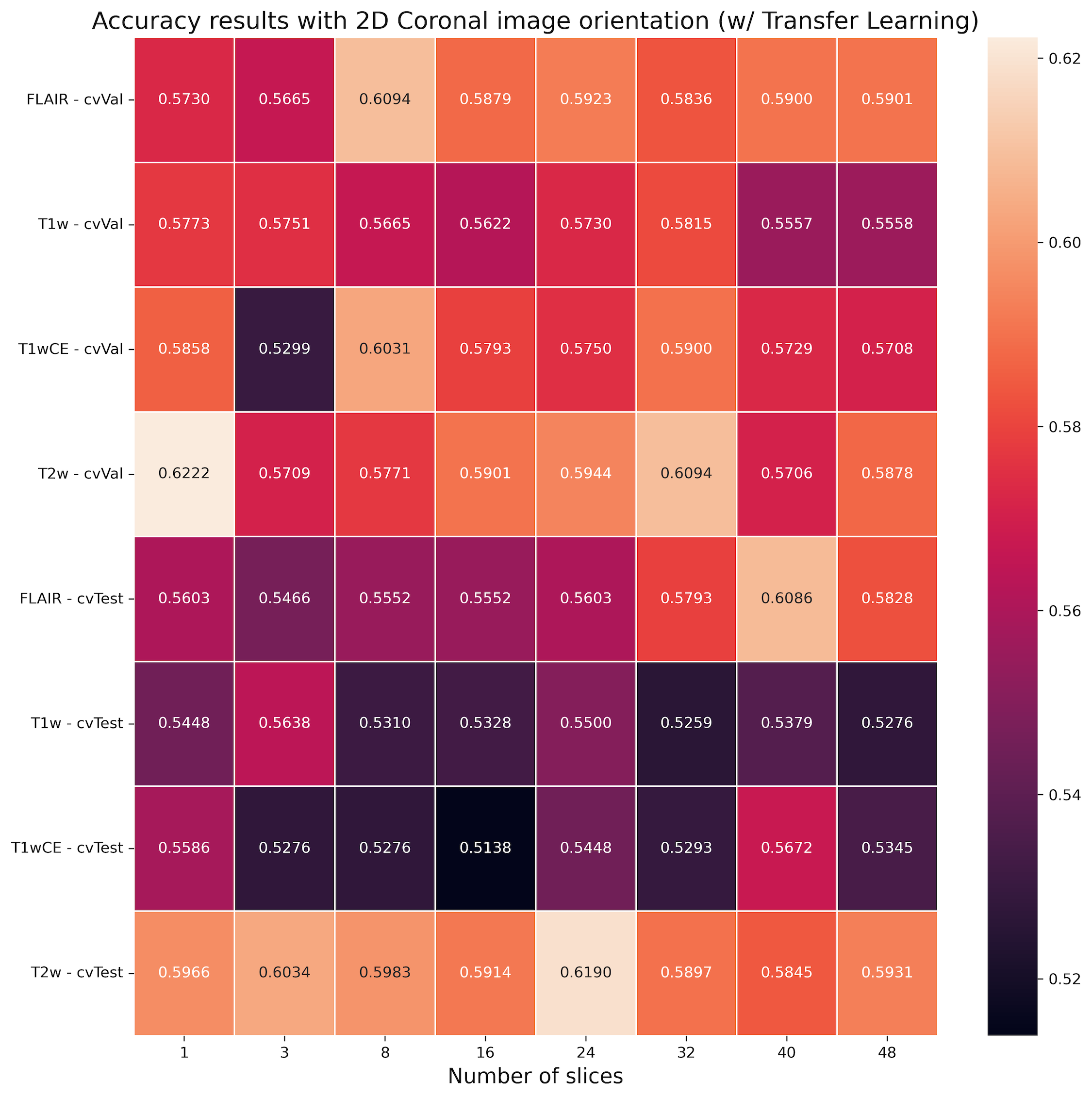

Supplement: S6 Fig — Training is done with 5-folds cross validation and results are averaged (for validation set and test set). (TIFF) [file pone.0351405.s006.tiff]

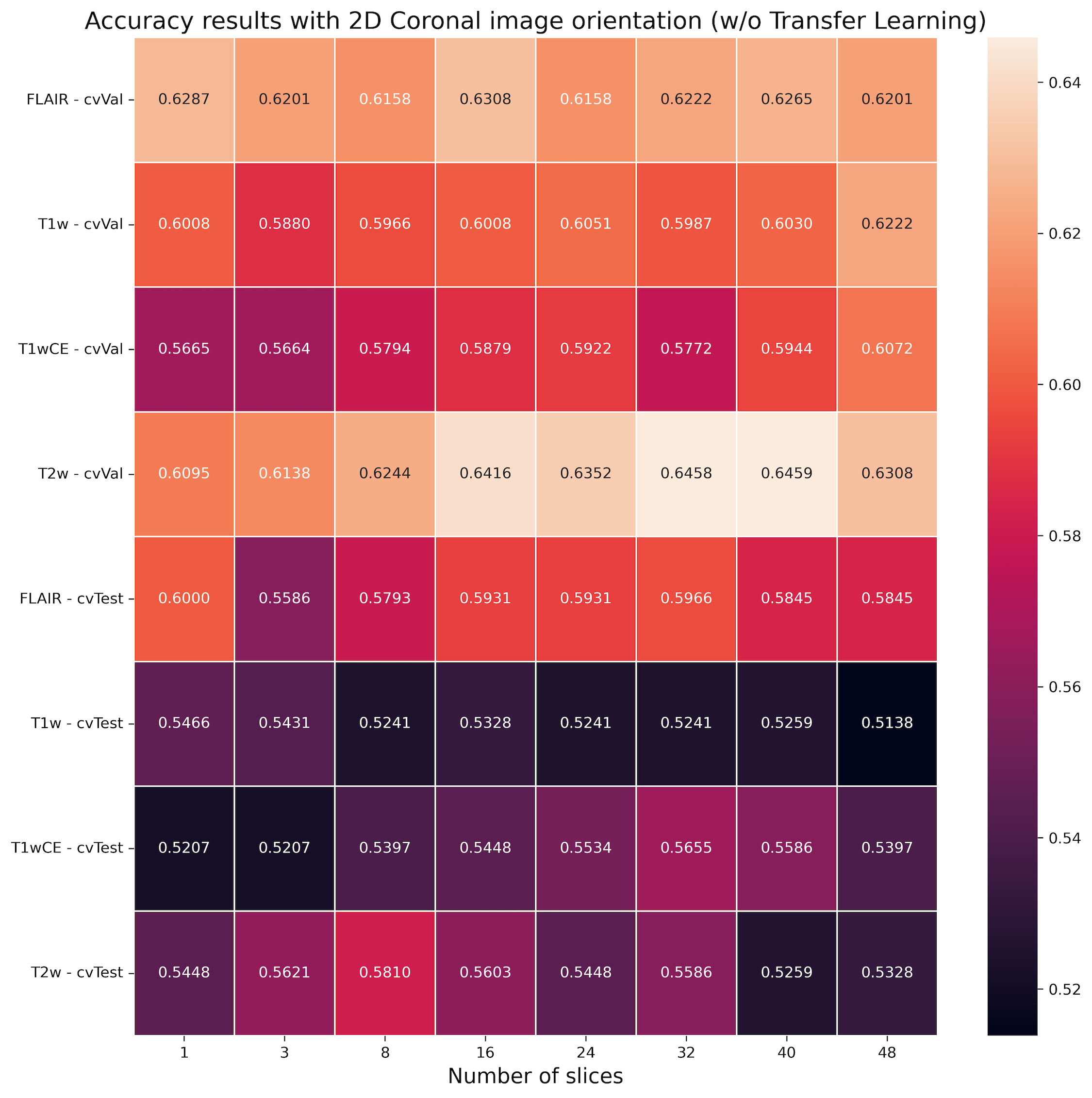

Supplement: S7 Fig — Training is done with 5-folds cross validation and results are averaged (for validation set and test set). (TIFF) [file pone.0351405.s007.tiff]

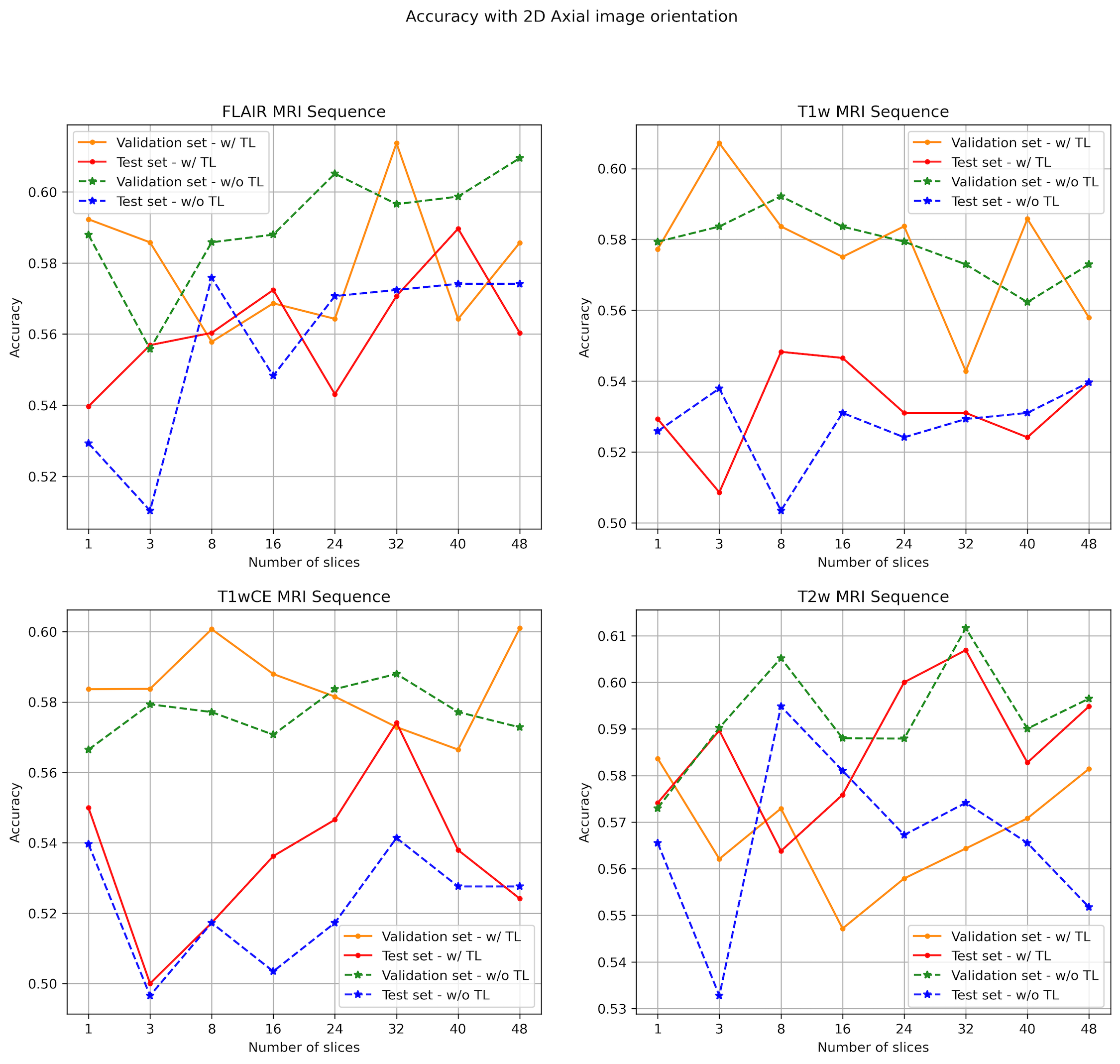

Supplement: S8 Fig — In each figure, there are results on validation set and test set with a model trained with Transfer Learning and a model trained without Transfer Learning. (TIFF) [file pone.0351405.s008.tiff]

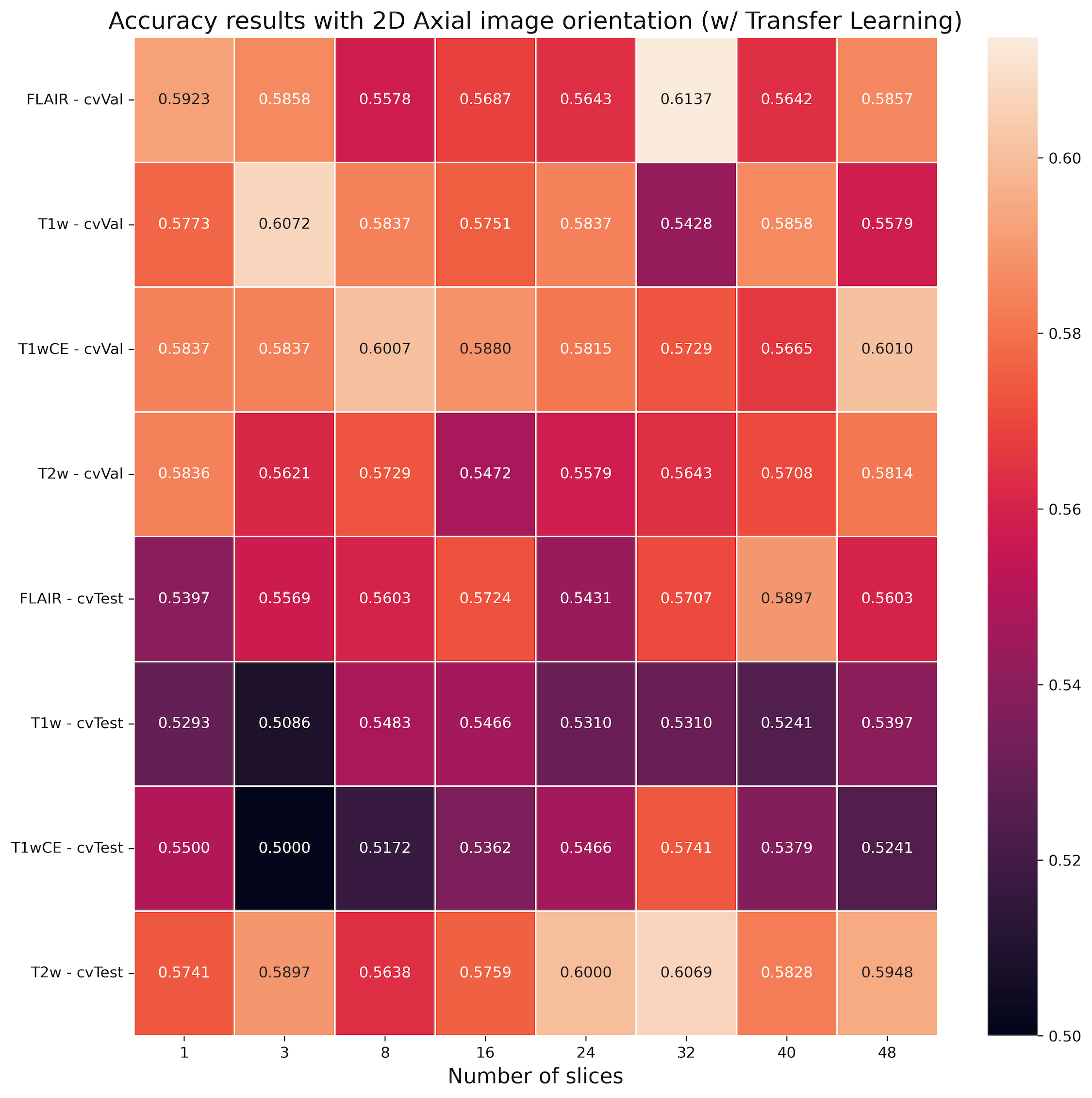

Supplement: S9 Fig — Training is done with 5-folds cross validation and results are averaged (for validation set and test set). (TIFF) [file pone.0351405.s009.tiff]

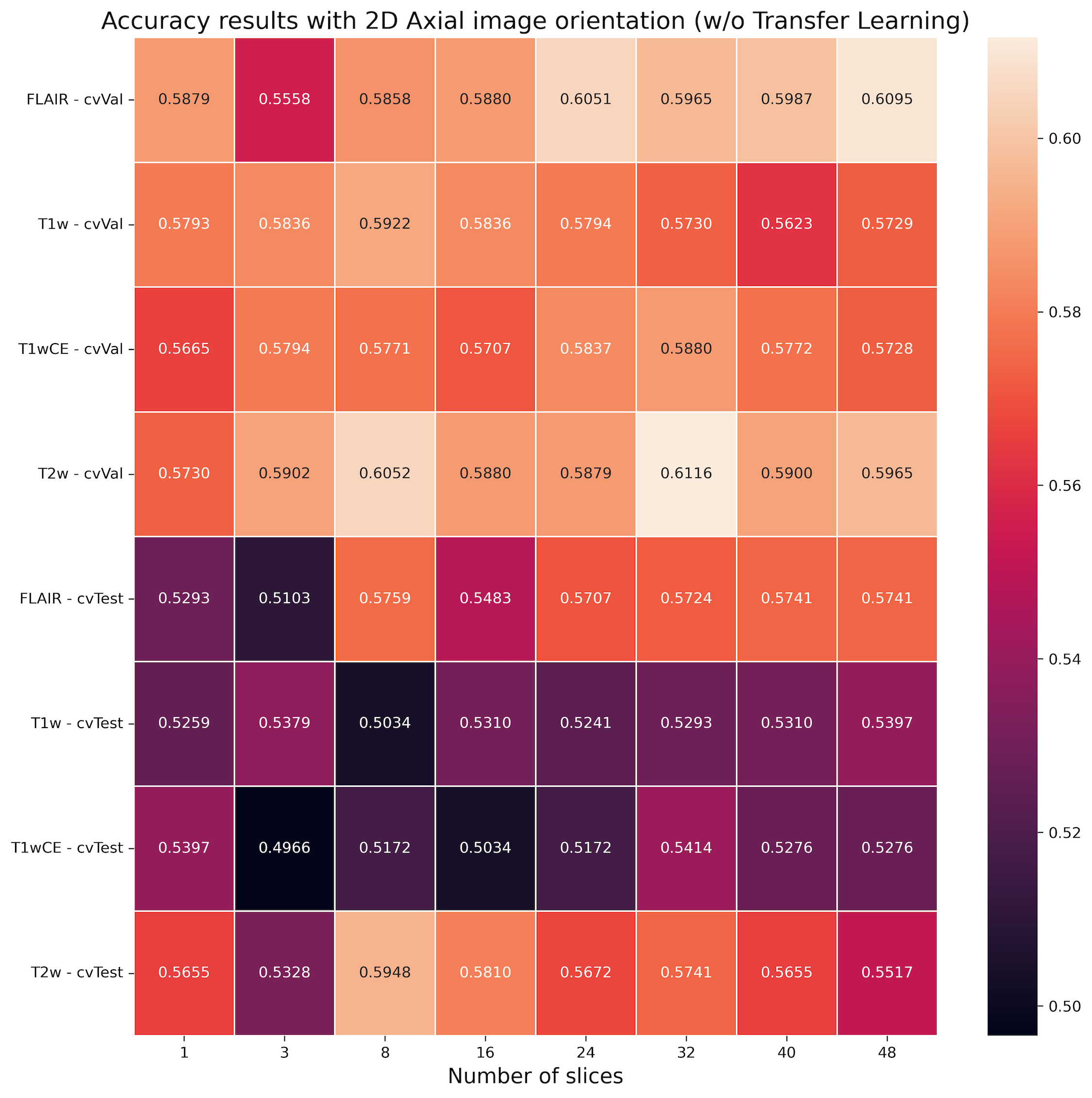

Supplement: S10 Fig — Training is done with 5-folds cross validation and results are averaged (for validation set and test set). (TIFF) [file pone.0351405.s010.tiff]

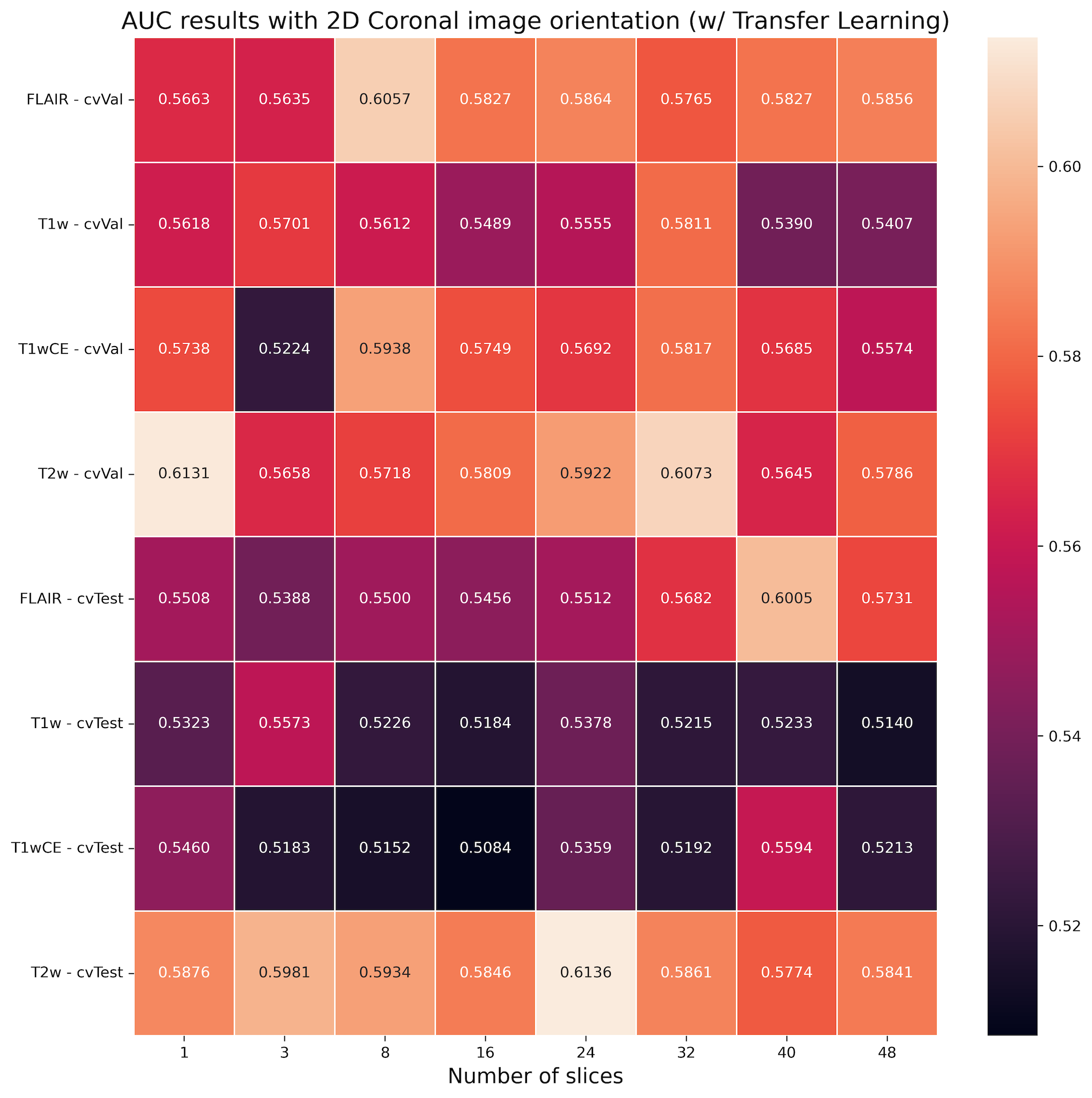

Supplement: S11 Fig — Training is done with 5-folds cross validation and results are averaged (for validation set and test set). (TIFF) [file pone.0351405.s011.tiff]

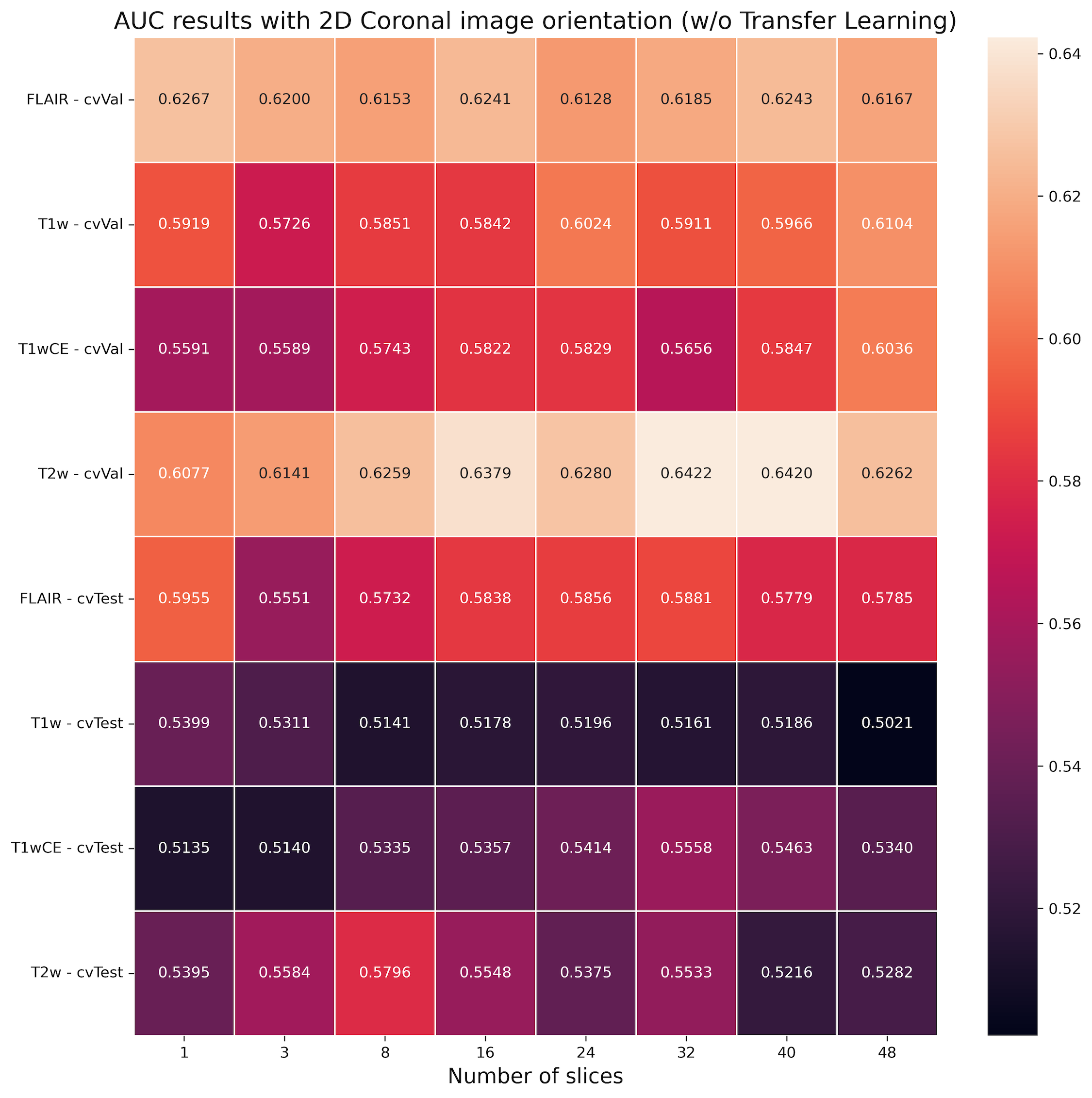

Supplement: S12 Fig — Training is done with 5-folds cross validation and results are averaged (for validation set and test set). (TIFF) [file pone.0351405.s012.tiff]
