# Supplementary material for: Unimodal vs. multimodal deep learning for non-invasive MGMT promoter methylation prediction in glioblastoma: A systematic evaluation on the BraTS 2021 dataset
Source: PLoS One. 2026 Jun 12;21(6):e0351405. doi: 10.1371/journal.pone.0351405 (PMC13262868; doi:10.1371/journal.pone.0351405)
Supplement: Attachment — Submitted filename: Response_to_Reviewers_MGMT_PLOSONE_v2.docx [file pone.0351405.s014.docx]

**Response to Reviewers**

*Unimodal vs. multimodal deep learning for non-invasive MGMT promoter methylation prediction in glioblastoma: a systematic evaluation on the BraTS 2021 dataset*

Submitted to PLOS ONE

**Response to Reviewer #1**

We thank Reviewer #1 for the positive assessment of the study's systematic design and for the detailed, constructive comments. We address each point below.

**Comment 1 — Slice selection methodology**

**Reviewer comment:**

*Unclear and insufficiently justified slice selection. The authors describe the number of slices used but do not specify clearly which slices from the volume are actually selected. This is a crucial point, especially with few slices: how do you ensure that at least one contains the tumor or an informative region? If the selected slices do not include the tumor, the prediction risks becoming essentially random or based on irrelevant signals.*

**Response:**

We thank the reviewer for raising this important methodological point. The slice selection strategy is described in the Methods section (Pre-processing subsection): slices are selected starting from the middle of the volume and expanding symmetrically outward. For example, with n=1 slice, the central slice is selected; with n=3, the central slice and one on each side; and so on. This center-out strategy is motivated by the fact that, in the BraTS 2021 dataset, MRI scans are skull-stripped and co-registered to a standard template, making the tumor region statistically likely to be near the center of the volume.

We fully acknowledge that this heuristic does not guarantee tumor coverage for all patients, particularly for small or peripherally located tumors, and that this constitutes a genuine methodological limitation. We have added a dedicated clarification in the Pre-processing subsection and strengthened the corresponding limitation discussion in the Study Limitations section. We note that this limitation is, in fact, consistent with the whole-brain, segmentation-free paradigm of this study, which deliberately avoids the use of tumor masks in order to evaluate the raw discriminative capacity of mpMRI sequences — a choice that is explicitly acknowledged as a limitation (see Absence of tumor segmentation, Study Limitations section).

**Manuscript changes:**

Pre-processing subsection: Added explicit description of the center-out slice selection strategy with the rationale based on co-registration to standard template. (l.175-178): “*For this study, the standardization is used with its parameters computed for each scan. In the final pre-processing step, a defined number of slices is extracted symmetrically around the central slice of the scan. When a single slice is selected, only the central one is retained; when multiple slices are chosen, the selection extends outward from the center in both directions.”*

Study Limitations section: The existing paragraph on 2D slice-based architecture is corrected to address slice selection uncertainty as a specific sub-limitation (l.492-493): “*A major limitation of this study is the absence of tumor region segmentation, both in slice selection and at the model input level.*”

**Comment 2 — Arithmetic errors in experiment count**

**Reviewer comment:**

*Inaccuracies in the total number of experiments. At line 313, the product 11×6×2×3 equals 396, but the text also mentions 369. Additionally, 1188 = 396×3 is reported, but the path to the total of 1,584 experiments is not shown precisely and transparently.*

**Response:**

The reviewer is correct. There are two distinct errors in the manuscript that we correct as follows:

• **Error 1:** "369" in the text is a typographical error. The correct product 11 × 6 × 2 × 3 = 396, and this is the correct value to report.

• **Error 2:** The figure of 1,584 total experiments cited in the Abstract corresponds to the sum of all unimodal and multimodal experiments but is an error: 396 (unimodal: 4 sequences × 8 slice counts × 2 TL conditions × 3 planes = 192) and 1,188 (multimodal with 3 fusion strategies: 396 × 3). The correct breakdown is: 192 (unimodal) + 1,188 (multimodal) = 1,380. This decomposition was not made explicit in the text, and we correct this.

We acknowledge that the figure of 1,584 given in the Abstract requires correction and precise justification, which we provide in the revised manuscript.

**Manuscript changes:**

Line 323: '369' corrected to '396'.

Abstract: The total experiment count is corrected, and the full arithmetic decomposition (unimodal + multimodal) is made explicit with a parenthetical breakdown (l.34): “*exploring 1,380 experimental configurations (unimodal: 192; multimodal: 1,188).*”

**Comment 3 — Reference numbering errors in Tables 2 and 3**

**Reviewer comment:**

*Inaccuracies in bibliographic references. In Table 2, "Robinet et al.24" and "Saeed et al.25" appear, but in the bibliography, [24] corresponds to Robinet et al. 2023 and [25] to Saeed et al. 2023. The same error repeats in Table 3.*

**Response:**

The reviewer is correct. The reference numbers in Tables 2 and 3 are misaligned with the bibliography. Upon verification, the correct reference numbers are [19] for Robinet et al. 2023 and [20] for Saeed et al. 2023 in the current bibliography numbering. We correct these reference numbers in both tables.

**Manuscript changes:**

Tables 2 and 3: Reference corrected from [24] to [19] for Robinet et al. and from [25] to [20] for Saeed et al. The full bibliography is verified for internal consistency.

**Comment 4 — Overconfident concluding statements**

**Reviewer comment:**

*Overly strong phrasing in concluding statements. The authors claim that (i) multimodality does not improve performance, (ii) the bottleneck is not the number of modalities but the quality/specificity of features, and (iii) T2w coronal should be prioritized for future data collection. These conclusions stem from a rather specific framework based on a single backbone family (VGG-16 from 2014) and relatively simple fusion strategies. The phrasing should be more cautious.*

**Response:**

We agree with the reviewer that these conclusions require more careful scoping. The study is explicitly designed as a systematic benchmark within a 2D VGG-16 framework, and the generalizability of these findings to other architectures such as 3D approaches, or more sophisticated fusion strategies cannot be claimed from these results alone. We have revised the relevant passages throughout the manuscript — in the Abstract, Discussion, and Conclusion — to systematically scope all three claims with the qualifier "within the tested 2D VGG-16 framework" or equivalent.

We note, however, that our finding regarding the multimodal performance ceiling is consistent with results reported by Saeed et al. (2023) using a broader set of architectures including ResNet-34 and EfficientNet-B1, suggesting that the limitation may extend beyond VGG-16 in this dataset. This convergent evidence is retained in the revised Discussion.

**Manuscript changes:**

Abstract (l.42): “*These findings suggest, for the tested framework in this study, that MGMT…*”

Discussion (l.405-407): *“A central and robust finding of this study is that combining multiple MRI sequences through any of the three fusion strategies — early, intermediate, or late — does not yield better-performing models than the best unimodal approach within the tested 2D VGG-16 framework.”*

Discussion (l.413-416): “The behavior observed in our model suggests that, at least in the present experimental setting, performance bottleneck appear to stem primarily from insufficient feature specificity and quality, particularly the absence of explicit tumor region localization, rather than from a lack of input modality diversity.”

Conclusion (l.554-555): “Based on our empirical findings, T2w coronal images appear to be more suited to develop a better model, …”

**Comment 5 — Single train/test split validation**

**Reviewer comment:**

*Weak validation framework. All experiments are evaluated on a single training/testing split, despite 5-fold cross-validation within training. Performances could thus be substantially influenced by the initial split's random seed. To demonstrate true robustness, a nested cross-validation or repetition over multiple splits would be preferable.*

**Response:**

We acknowledge this limitation. The use of a single 80/20 train-test split does introduce sensitivity to the random seed of that partition, and nested cross-validation would in principle provide a more robust variance estimate. We fully recognize this as a methodological weakness.

However, we respectfully note that repeating all experiments over multiple splits is computationally prohibitive given the scale of this study: 1,380 trained models already represent a substantial computational investment because it took approximately 1 month of computing to obtain the results in this study despite optimization and parallelization. Multiplying this by N independent splits (e.g., N=5 for a nested scheme) would require a fivefold increase in compute. This constraint is acknowledged transparently in the Study Limitations section. We note furthermore that the single-split design is consistent with the majority of comparable published benchmarks on this dataset (including the BraTS 2021 challenge itself, Kim et al. 2023, and Robinet et al. 2023), making our results at least comparably constrained.

We have strengthened the limitations section to make this constraint explicit, noted the risk of split-dependent variance, and added the random seed of the split to the Methods section to ensure reproducibility.

**Manuscript changes:**

Methods (l.126-127): “*with a ratio 8:2 with respect to the initial distribution with a random seed for reproducibility.”*

Study Limitations section: New paragraph explicitly addressing the single-split design, the associated risk of seed-dependent variance, and the computational rationale for the chosen design (l.514-519): “*Single split design. The dataset was partitioned into training and test sets using a fixed random seed to ensure reproducibility. While this approach guarantees consistent splits across runs, it introduces a risk of seed-dependent variance, which represents a less robust alternative to methodologies such as nested cross-validation. However, the current pipeline already operates near its computational limits despite parallelization and runtime optimizations. Expanded computational capacity would be a prerequisite for moving toward a more reliable evaluation strategy.*”

**Comment 6 — Comparability of state-of-the-art tables**

**Reviewer comment:**

*Comparisons with state-of-the-art not always correct or fair. The comparison tables mix studies on different datasets and test sets. As presented, this risks being incorrect or at least misleading. The limits of comparability need clearer explanation.*

**Response:**

The reviewer raises a valid and important point. The limitations of these comparisons are already partially acknowledged in the tables footnotes ("* indicates that the results are difficult to compare"). The paragraph before the Table 2 is revised and a paragraph before Table 3 is added to systematically enumerates the sources of non-comparability: different datasets (BraTS 2021 public vs. private institutional cohorts) and different evaluation protocols (test set vs. cross-validation only). The term ‘comparison’ is replaced by ‘contextualization against literature’ in the text to better reflect the intended purpose of these tables.

**Manuscript changes:**

Table 2: The explanation is already in the footnote in the table and in the paragraph before the table (l.300-303): “*Table 2 allows a contextualization of our best results obtained with the unimodal approach against the literature. The test set used by the competition winner is not available to us. The study by Robinet et al. uses a private dataset, and Saeed et al. does not have a test set since they use cross-validation.”*

Table 3: The footnote is already present in the initial manuscript, and a paragraph is added in the revised manuscript (l.351-353): “*Results in Table 3 are accompanied by multimodal results found in the state-of-the-art. In the study of Robinet et al., they use a private test set not accessible for us. Saeed et al. uses cross-validation to train their model; therefore, they do not have any results on a test set.*”

**Comment 7 — Terminological inconsistencies**

**Reviewer comment:**

*Inconsistent terminology. Terminology is not fully uniform in several places throughout the manuscript. A stylistic and terminological review would improve overall clarity.*

**Response:**

We thank the reviewer for this observation. A full terminological audit of the manuscript has been conducted. Key inconsistencies identified and corrected include:

(1) 'without RL' corrected to 'without TL' (line 328, noted also by Reviewer #2);

(2) inconsistent capitalization of 'Coronal', 'Sagittal', and 'Axial' when used as plane descriptors (standardized);

**Manuscript changes:**

Full manuscript: Terminological standardization as described above. Line 328: 'RL' corrected to 'TL'.

**Comment 8 — Inconsistency between Table 2 and text (1 slice vs. 24 slices)**

**Reviewer comment:**

*Inconsistency between Table 2 and text on the best model. The text states that for the best model with transfer learning in the coronal plane, 'the best model is obtained by using T2w images with only 1 slice,' with validation accuracy 0.6222 and test accuracy 0.5966. However, Table 2 shows the best TL model using 24 slices.*

**Response:**

The reviewer is correct. There is an inconsistency between the body text and Table 2. The text correctly describes the best TL model with T2w coronal as the 1-slice configuration (validation accuracy 0.6222, test accuracy 0.5966).

The 2^nd^ line in Table 2 mistakenly showed the model with the best performance on the test set. The row is corrected to display the model with the best performance on validation set and with TL.

**Manuscript changes:**

Table 2, row 'VGG-16 This study (TL = Yes)': Results with the correct model is shown.

**Comment 9 — Overconfident recommendation on T2w coronal**

**Reviewer comment:**

*Overly strong discussion of T2w coronal. The observation is interesting, but the result remains highly dataset-specific and architecture-specific. The recommendation to prioritize T2w coronal in future data collection should be expressed more cautiously.*

**Response:**

We fully agree. This point is closely related to Comment #4, and the same revision applies. The recommendation regarding T2w coronal acquisitions is now explicitly qualified as a dataset-specific and framework-specific empirical observation, contingent on the 2D VGG-16 architecture and the BraTS 2021 dataset characteristics. The revised phrasing reads: 'Within the tested 2D VGG-16 framework on the BraTS 2021 dataset, T2w coronal images were consistently associated with the best-performing configurations. This observation may reflect dataset-specific imaging properties and should not be generalized without validation on independent cohorts and architectures.'

**Manuscript changes:**

Abstract (l.44-45): “… *T2w coronal acquisitions could be more interesting in future data collection efforts.*”

Discussion (l.390-393): “*'Within the tested 2D VGG-16 framework on the BraTS 2021 dataset, T2w coronal images were consistently associated with the best-performing configurations. This finding may reflect dataset-specific imaging properties and should not be generalized without validation on independent cohorts and architectures*.”

Conclusion (l.554-555): “*Based on our empirical findings, T2w coronal images appear to be more suited to develop a better model, …*”

**Response to Reviewer #2**

We thank Reviewer #2 for the thorough and rigorous evaluation. The reviewer correctly identifies several methodological limitations, and we respond to each point below with a combination of manuscript revisions and, where appropriate, scientific justification of the study design choices.

**Comment 1 — Scientific objective and clinical overreach**

**Reviewer comment:**

*The overall scientific objective remains insufficiently defined and internally inconsistent. The authors state that their primary aim is not to propose a new architecture but rather to characterize optimal input configurations (lines 113-116). However, they simultaneously derive strong clinical recommendations, such as prioritizing T2-weighted coronal acquisitions (lines 42-44, 499-500). This discrepancy between an exploratory methodological study and deterministic clinical conclusions is problematic.*

**Response:**

We agree entirely. The manuscript's primary contribution is a systematic benchmark study with a negative result: no configuration tested on the BraTS 2021 dataset reaches clinically actionable performance. The language in the Abstract and Conclusion was inconsistently calibrated, with some passages adopting a stronger prescriptive tone than the data support. We have revised the Abstract, Introduction (objective statement), and Conclusion to consistently frame the study as an exploratory benchmark. The T2w coronal observation is now presented as an empirical finding deserving further investigation rather than a clinical recommendation.

**Manuscript changes:**

Abstract (l.42-45): “*These findings suggest, for the tested framework in this study, that MGMT methylation status prediction from mpMRI remains fundamentally constrained by dataset heterogeneity and size, irrespective of modality combination strategy, and that T2w coronal acquisitions could be more interesting in future data collection efforts.*”

Introduction (l.113-118): “*Our primary aim is not to propose a new architecture, but to systematically characterize which input features — modality, plane, slice count, and fusion strategy — yield the best-performing and most generalizable models, within the tested 2D VGG-16 framework, for MGMT promoter methylation prediction, thereby offering preliminary insights that could be more efficient for future data collection and model development in this clinically critical task.*”

Conclusion (l.554-555): “*Based on our empirical findings, T2w coronal images appear to be more suited to develop a better model, …*”

**Comment 2 — Dataset split and potential test set leakage**

**Reviewer comment:**

*The dataset is split into an 80/20 train-test partition prior to applying five-fold cross-validation. This approach is suboptimal and introduces a risk of bias, as it does not constitute a truly independent test evaluation and may lead to implicit information leakage or indirect model tuning on the test set. A nested cross-validation scheme or an external validation cohort would have been more appropriate.*

**Response:**

We thank the reviewer for this methodological remark. We wish to clarify the exact design to address the leakage concern directly: the 80/20 split is performed once before any model training or hyperparameter selection. The 20% test set is strictly held out and never used during training, fold construction, or any model selection decision. The 5-fold CV is applied exclusively within the 80% training partition. Model selection (e.g., best configuration) is made on the validation folds, and the test set is used solely for final evaluation of the selected configurations. This design does not involve any iterative or indirect tuning on the test set; it is a standard stratified hold-out + k-fold design widely used in the literature on this specific dataset (Kim et al. 2023; Saeed et al. 2023).

We fully acknowledge that nested cross-validation would provide a less biased estimate of generalization performance, as noted by the reviewer. However, as argued in response to Reviewer #1 Comment #5, the computational cost of repeating 1,380 experiments across nested folds is prohibitive in the context of this study. We have improved the clarity of the Methods description to eliminate any ambiguity about the absence of test set leakage.

**Manuscript changes:**

Methods (l.189-192): “*A random set of 20% of patients (test set) is held out and never used during training or any model selection decision, thus avoiding any risk of data leakage. The test set is strictly used to evaluate the generalization capacity of the model. A 5-fold cross-validation is exclusively applied on the remaining 80% training partition.*”

Study Limitations section (l.514-519): “*Single split design. The dataset was partitioned into training and test sets using a fixed random seed to ensure reproducibility. While this approach guarantees consistent splits across runs, it introduces a risk of seed-dependent variance, which represents a less robust alternative to methodologies such as nested cross-validation. However, the current pipeline already operates near its computational limits despite parallelization and runtime optimizations. Expanded computational capacity would be a prerequisite for moving toward a more reliable evaluation strategy.*”

**Comment 3 — 2D slice-based analysis vs. volumetric 3D modeling**

**Reviewer comment:**

*The use of 2D slice-based analysis instead of volumetric 3D modeling represents a significant limitation, as it disregards essential spatial information inherent to MRI data. This is particularly critical in glioblastoma, where tumor heterogeneity and infiltration patterns are inherently three-dimensional.*

**Response:**

We respectfully argue that the 2D slice-based design is not a flaw but a deliberate and scientifically motivated methodological choice, for the following reasons:

**1. Study objective:** The primary aim of this study is to conduct a controlled, exhaustive combinatorial evaluation of input configurations (sequence, plane, slice count, fusion strategy) within a fixed architecture. Using a 2D backbone provides a computationally tractable framework for this 1,380-configuration exploration. A 3D architecture would multiply memory and training costs by an order of magnitude, making the same exhaustive search infeasible.

**2. Literature precedent:** 2D CNN approaches remain used in radiogenomics benchmarks on this dataset (Saeed et al. 2023). The 3D-ResNet10 winner of the BraTS 2021 challenge achieved an AUC of only 0.622, a performance not substantially different from our best 2D model, and degraded to 0.562 on external validation (Kim et al. 2023). This suggests that the performance ceiling is not primarily a function of 2D vs. 3D modeling.

**3. Acknowledged limitation:** The 2D nature of the backbone is already explicitly acknowledged in the Study Limitations section as a direction for future work. We reinforce this acknowledgment in the revised manuscript.

**Manuscript changes:**

Methods (l.211-214): Added explicit justification for the 2D design choice in the context of the exhaustive combinatorial search objective: “*The primary aim of this study is to conduct a controlled, exhaustive combinatorial evaluation of input configurations (sequence, plane, …) without a variance caused by a different architecture. Using a 2D backbone instead of a 3D model provides a more computationally tractable framework.*”

Study Limitations section (l.502-504): Strengthened the 3D limitation paragraph with reference to the BraTS challenge 3D winner and its external validation performance: “*It is noteworthy that the BraTS 2021 3D model winner exhibited a decrease in performance when evaluated on an independent dataset (Kim et al. [29]).*”

**Comment 4 — Absence of tumor segmentation**

**Reviewer comment:**

*The study does not incorporate tumor segmentation, instead relying on whole-brain images. This decision substantially reduces the signal-to-noise ratio and likely dilutes any biologically relevant features associated with MGMT status. While the authors acknowledge this as a limitation, it should be considered a fundamental methodological flaw rather than a secondary issue.*

**Response:**

We partially agree with the reviewer's characterization. The absence of tumor segmentation is indeed a substantial limitation, and we accept that our original presentation understated its significance. We elevate this point to the primary limitation in the revised Study Limitations section.

However, we respectfully maintain that this choice is not a methodological flaw in the context of the study's explicit objective. This study is designed to characterize the discriminative capacity of raw mpMRI sequences without any domain knowledge (such as tumor masks), as a deliberate and reproducible baseline that (1) avoids the dependency on a separate segmentation model, (2) is directly comparable across the full combinatorial space of configurations, and (3) provides a clear lower bound against which segmentation-guided approaches can be measured. The finding that even whole-brain 2D approaches reach ~0.64 AUC — matching or exceeding 3D models in some configurations — is itself informative.

**Manuscript changes:**

Study Limitations section (l.492-498): The segmentation limitation is elevated to primary status. Added explicit statement that tumor-guided approaches using BraTS segmentation masks represent the most directly actionable extension of this work: “*A major limitation of this study is the absence of tumor region segmentation, both in slice selection and at the model input level.*” … “*Integration of explicit tumor segmentation—whether manual or automated (e.g., via U-Net-based approaches)—represents a primary avenue for extending this work, with the potential to enhance the signal-to-noise ratio and improve model performance.*”

**Comment 5 — Choice of VGG-16 architecture**

**Reviewer comment:**

*The choice of model architecture is also insufficiently justified. The exclusive use of VGG-16, a relatively outdated architecture in the context of modern medical imaging, is not adequately motivated, and no comparison with more advanced models such as 3D CNNs, EfficientNet variants, or transformer-based approaches is provided. This limits the study's relevance to the current state of the field.*

**Response:**

We respectfully disagree with the characterization of VGG-16 as incompatible with the study's scientific contribution, and we argue as follows:

**1. Role in the study:** VGG-16 serves as a controlled, fixed architectural backbone. The study is not a model comparison study; it is a systematic input-configuration study. Holding the architecture constant is a deliberate design choice that allows the effect of input variables (sequence, plane, slice count, fusion strategy) to be isolated from architectural variation. Introducing multiple architectures would confound the analysis.

**2. Established relevance in the field:** VGG-16 remains a widely used backbone in medical imaging benchmarks ([Djaroudib et al. 2024](https://www.mdpi.com/2076-3417/14/17/7447), [Loganayagi et al. 2025](https://www.sciencedirect.com/science/article/abs/pii/S0168165625001257)). Furthermore, Saeed et al. (2023) demonstrated that EfficientNet-B1 — a more modern architecture — achieved only marginally higher AUC (0.63) than ResNet-34 (0.61) on the same BraTS dataset. In our study, VGG-16 achieved similar results. This further supports the conclusion that architecture choice is not the primary determinant of performance on this dataset.

**3. Computational tractability:** The current pipeline with only VGG-16 is already time-consuming (approximately 1 month) despite parallelization and optimization. Running 1,380 experiments with more models such as transformer-based architectures (e.g., ViT, Swin Transformer) would be computationally infeasible within the resource envelope of this study. We are transparent about this constraint.

We have strengthened the justification of the VGG-16 choice in the Methods section (following comment #3 from Reviewer #2) and added a note in the Discussion that the study's conclusions are scoped to the VGG-16 backbone, consistent with revisions made in response to Comments #4 and #9 of Reviewer #1.

**Manuscript changes:**

Methods (l.211-214): Expanded justification for VGG-16 as a controlled baseline backbone for exhaustive combinatorial search: “*The primary aim of this study is to conduct a controlled, exhaustive combinatorial evaluation of input configurations (sequence, plane, …) without a variance caused by a different architecture. Using a 2D backbone model such as VGG-16 instead of a 3D model provides a more computationally tractable framework.*”

Study limitations (l.524-529): A new paragraph to explicitly discuss that the results are scoped to the VGG-16 backbone: “*Single-framework evaluation. The observations reported in this study are based on a single architectural framework (2D VGG-16), making it challenging to determine whether the observed effects are dataset-related or framework-dependent. While findings in prior work tends to suggest a stronger influence of dataset characteristics, this remains inconclusive in the present setting. Reproducing the pipeline across more recent and diverse architectures would therefore be necessary to assess the robustness and generality of these findings.*”

Discussion: Explicit acknowledgment that the conclusions are VGG-16-specific made to respond Comment #4 and #9 from Reviewer #1. (l.365-367; 390-393; 405-416)

**Comment 6 — Insufficient statistical analysis**

**Reviewer comment:**

*The analysis is notably incomplete. The authors report average performance metrics across cross-validation folds but do not provide measures of variability such as standard deviations or confidence intervals. Furthermore, no statistical testing is performed, despite claims that no meaningful differences exist between fusion strategies. The evaluation is restricted to accuracy and AUC, without consideration of calibration, precision-recall analysis, or decision curve analysis.*

**Response:**

We fully accept this critique. The absence of variability measures (standard deviations across folds) is a genuine weakness that we correct in the revised manuscript. For all reported mean accuracy and AUC values across the 5-fold CV, we now report mean ± standard deviation. This applies to the key results tables (Tables 2 and 3) and the corresponding text passages.

Regarding formal statistical testing between fusion strategies: we agree that the claim 'no significant ranking emerged' requires either a statistical test or more careful phrasing. In the revised manuscript, we have removed inferential wording about the differences in fusion strategies when this was not formally tested and replaced it with more descriptive language.

Regarding additional metrics (precision-recall, calibration, decision curve analysis): we note that precision-recall curves are already partially addressed through sensitivity/specificity reporting (Table 4). Given the study's exploratory framing and the number of evaluated configurations, full calibration and decision curve analyses for all 1,380 models are not feasible. However, we add F1-score and precision alongside sensitivity/specificity for the best-performing models in Tables 2 and 3, and we acknowledge the absence of calibration analysis as a limitation.

**Manuscript changes:**

Tables 2 and 3: Added mean ± SD for all CV-reported metrics.

Results text: Standard deviations added throughout.

Discussion (l.420-425): Paragraph revised to remove the formal comparison: “*In this study, the best-performing models obtained for each evaluated fusion strategy reached peak performance levels similar to those observed with the unimodal approach, despite the extensive set of experiments conducted using a common backbone architecture. This observation may indicate that the limited benefit of multimodality in this context is not primarily attributable to architectural design choices but could instead reflect underlying data-related constraints.*”

Discussion (l.467-479): Added a paragraph with precision and F1 score metrics: “*Precision and F1 score are another metrics to further the investigation about the clinical relevance of our models. Precision measures the proportion of correctly identified positive cases among all samples predicted as positive, thereby reflecting the model’s ability to limit false-positive predictions. A high precision indicates that positive predictions are highly reliable. The F1-score, defined as the harmonic mean of precision and sensitivity, provides a single summary metric that balances the trade-off between correctly identifying positive cases and avoiding missed detections. High F1 score implies that the model can both detect methylated cases effectively and maintain robustness against false alarms. The model trained with 32 slices of T2w coronal images without TL have a precision of 0.62 and a F1 score of 0.67 on the validation set and 0.57 and 0.61 respectively on the test set. For the model initialized with TL and trained with 1 slice of T2w coronal image, the precision is 0.62 and the F1 score is 0.68 on the validation set. On the test set, the metrics are 0.59 and 0.66. Table 5 shows the precision and F1 score for the best multimodal approach in the same manner of Table 4.*” Table 5 is added with precision and F1 scores for multimodal models.

Study Limitations (l.509-513): Added note on absence of calibration analysis: “*Limited evaluation metrics. The evaluation primarily relies on accuracy and AUC, without incorporating calibration or decision curve analyses. Although additional metrics (e.g., precision and F1-score) were reported for the best-performing models, the absence of calibration assessment limits insight into the reliability of predicted probabilities. This aspect should be addressed in future work to support more comprehensive model evaluation.”*

**Comment 7 — Interpretation of performance ceiling**

**Reviewer comment:**

*The authors attribute the observed performance ceiling primarily to dataset-related factors such as label noise and heterogeneity, yet alternative explanations — most notably the limitations of the chosen methodology — are not sufficiently explored. The claim that MGMT prediction is 'fundamentally constrained' by dataset properties is therefore speculative and not convincingly supported.*

**Response:**

We accept that the language 'fundamentally constrained' overstates the certainty of our interpretation. The performance ceiling could in principle reflect dataset properties (label noise, heterogeneity), methodological limitations (2D architecture, no segmentation), or a combination of both. We cannot distinguish between these explanations from the current data alone.

We have revised the relevant passages to present dataset-related and methodology-related explanations as co-existing, non-exclusive hypotheses. We explicitly note that the strongest evidence for a dataset-level ceiling comes from external sources — specifically the findings of Kim et al. (2023), who showed that a 3D-ResNet trained on BraTS achieves only AUC 0.562 on an independent 400-patient external cohort, suggesting that the ceiling persists even with a more powerful architecture on a truly independent test set. This external evidence strengthens the dataset-ceiling hypothesis beyond what our own results alone could support.

**Manuscript changes:**

Discussion (l.365-377): Revised to present dataset-level and methodology-level explanations as co-existing hypotheses. The Kim et al. (2023) external validation finding is now explicitly mobilized as supporting evidence for the dataset-level ceiling hypothesis: “*Based on our results, the hypothesis of a performance ceiling can be made, however it is unclear if it is primarily dataset-related or methodology-related. A safe assumption is to put responsibility on both aspects.*” … “*Kim et al. [29] …. These results tend to shift the responsibility of the performance ceiling to be primarily dataset related.”*

**Comment 8 — Formal inconsistencies and typographical errors**

**Reviewer comment:**

*The reported patient numbers are not consistently described (lines 32 vs. 121-122), and there are minor errors including inconsistent reporting of model counts (line 313) and typographical inaccuracies (e.g., 'trained without RL' instead of TL, line 318).*

**Response:**

All formal inconsistencies identified by the reviewer have been corrected:

• **Patient count:** Line 32 reports 582 patients; lines123-124 report the initial size of the dataset (585 patients) before excluding 3 patients due to some data error. The effective size of the dataset used in this study is 582. The distinction is now made explicit.

• **Line 313 model count:** Corrected as described in Response to Reviewer #1, Comment #2.

• **Line 318 'RL' → 'TL':** Corrected.

**Manuscript changes:**

Lines 123-125: “*The usable dataset is initially composed of 585 instances, but 3 patients must be removed due to some discrepancies (patients: 00109, 00123, 00709) – 306 methylated patients and 276 unmethylated patients making an effective dataset of 582 patients.*”

Line 323: Corrected as per Reviewer #1 Comment #2.

Line 328: 'RL' corrected to 'TL'. Full terminological audit applied throughout (see also Reviewer #1 Comment #7).
