# Supplementary material for: Unimodal vs. multimodal deep learning for non-invasive MGMT promoter methylation prediction in glioblastoma: A systematic evaluation on the BraTS 2021 dataset
Source: PLoS One. 2026 Jun 12;21(6):e0351405. doi: 10.1371/journal.pone.0351405 (PMC13262868; doi:10.1371/journal.pone.0351405)
Supplement: Attachment — Submitted filename: 23052026_Response_to_Reviewers_v2.docx [file pone.0351405.s015.docx]

**Response to Reviewer — Minor Revision**

*Unimodal vs. multimodal deep learning for non-invasive MGMT promoter methylation prediction in glioblastoma: a systematic evaluation on the BraTS 2021 dataset*

Submitted to PLOS ONE

**Response to Reviewer #2**

We thank Reviewer #2 for the thorough and constructive re-reading of the revised manuscript. We note with appreciation that the reviewer considers the work substantially improved and acceptable for publication after minor editorial corrections. We address the four identified inconsistencies below.

**General remark.** The reviewer correctly observes that several formulations described as revised in the rebuttal letter were not consistently implemented in the uploaded manuscript. We have now verified the full manuscript against every stated commitment in the response letter and corrected all outstanding discrepancies. The five specific issues are detailed below.

**Inconsistency 1 — Abstract — “Fundamentally constrained”**

**Reviewer observation:**

*The Abstract still contains relatively strong wording such as “fundamentally constrained” [...] despite the authors indicating that these conclusions had been reformulated more cautiously.*

**Analysis:**

In the rebuttal letter (Response to Reviewer #2, Comment 7), we committed to presenting the performance ceiling as a co-hypothesis between dataset-related and methodology-related factors. The term *“fundamentally constrained”* overstates the certainty of our interpretation.

The Discussion section was correctly revised to reflect this nuance (co-hypothesis framing, Kim et al. external evidence). However, the Abstract retained the original phrasing. The addition of the qualifier *“, for the tested framework in this study,”* introduced in the revised manuscript partially addresses the scoping concern but does not resolve the overstatement carried by *“fundamentally constrained”.* We therefore revise this phrase in the Abstract to achieve full consistency with the Discussion.

The term of *“fundamentally constrained”* is replaced with *“appears primarily limited”* to better align the Abstract with both the Discussion and the Introduction. The qualifier *“appears primarily”* acknowledges that methodological factors may also contribute, which is consistent with our stated position in the rebuttal.

**Manuscript change:**

Abstract (l.43): “*fundamentally constrained*” → “*appears primarily limited*”.

**Inconsistency 2 — Abstract — T2w coronal phrasing**

**Reviewer observation:**

*The Abstract still contains relatively strong wording such as [...] “should be prioritized in future data collection efforts” despite the authors indicating that these conclusions had been reformulated more cautiously.*

**Analysis:**

The rebuttal letter committed to replacing the prescriptive phrasing *“should be prioritized”* with a dataset-specific empirical observation. The revised manuscript introduced *“could be more interesting”* (Abstract) and *“T2w coronal images appear to be more suited to develop a better model”* (Conclusion). While these represent an improvement over the original, the Abstract phrasing *“could be more interesting”* remains imprecise and does not adequately convey the conditionality and dataset-specificity of the observation. We replace it with a formulation that is explicitly scoped and empirically grounded, consistent with the Discussion wording already in the revised manuscript.

**Manuscript changes:**

Abstract (l.44-45): “*and that T2w coronal acquisitions could be more interesting in future data collection efforts*” → “*In this setting, T2w coronal images were associated with the most competitive configurations and may warrant further investigation in future studies*”.

**Inconsistency 3 — Introduction — Experiment count inconsistency (“over 1,300” vs. 1,380)**

**Reviewer observation:**

*The total number of experimental configurations in the Abstract still appears inconsistent with the corrected arithmetic described in the rebuttal letter.*

**Analysis:**

The rebuttal letter committed to correcting the total experiment count and providing the explicit breakdown 192 (unimodal) + 1,188 (multimodal) = 1,380. The Abstract was correctly updated to *“1,380 experimental configurations (unimodal: 192; multimodal: 1,188)”*. However, the Introduction paragraph (objective statement) still reads *“over 1,300 model configurations,”* which is inconsistent with 1,380 and contradicts the explicit corrected figure. We replace this with the exact figure.

**Manuscript change:**

Introduction (l.108-109): “*we trained and evaluated over 1,300 model configurations*” → “*we trained and evaluated 1,380 model configurations (192 unimodal; 1,188 multimodal)*”.

**Inconsistency 4 — Introduction — Residual clinical tone (“clinically critical task”)**

**Reviewer observation:**

*Some phrasing in the Introduction continues to imply stronger clinical applicability than intended according to the revised study framing.*

**Analysis:**

The rebuttal letter (Response to Reviewer #2, Comment 1) committed to reframing the study consistently as an exploratory benchmark and removing prescriptive clinical language from the Introduction objective statement. The revised manuscript introduced the explicit scope qualifier *“within the tested 2D VGG-16 framework”* and replaced *“should be prioritized”* with softer formulations. However, the concluding clause of the objective statement in the Introduction (*“thereby offering preliminary insights that could be more efficient for future data collection and model development in this clinically critical task”*) retains a prescriptive tone inconsistent with the exploratory benchmark framing. Specifically, *“clinically critical task”* implies the models could be clinically deployed, which is explicitly contradicted by the Study Limitations and Discussion sections. We revise this clause to neutral, methodologically scoped language.

**Manuscript change:**

Introduction (l.116-118): *“… thereby offering preliminary insights that could be more efficient for future data collection and model development in this clinically critical task.”* → “*Accordingly, the present study should be interpreted as an exploratory benchmark intended to inform subsequent methodological work and future data collection strategies*”.

**Inconsistency 5 — Results — Residual internal inconsistency**

**Analysis:**

During the final consistency check, we identified and corrected one remaining internal inconsistency in the manuscript itself: the caption associated with the best coronal T2w configuration in Figure 5 still referred to 40 slices, whereas the corrected body text indicated 32 slices. This discrepancy has now been corrected.

**Manuscript change:**

Results (l.275): *“… T2w modality and 40 slices …”* → “*… T2w modality and 32 slices …*”.
