# Supplementary material for: Unimodal vs. multimodal deep learning for non-invasive MGMT promoter methylation prediction in glioblastoma: A systematic evaluation on the BraTS 2021 dataset
Source: PLoS One. 2026 Jun 12;21(6):e0351405. doi: 10.1371/journal.pone.0351405 (PMC13262868; doi:10.1371/journal.pone.0351405)
Supplement: Peer review history

# Peer review: Decision Letter 0

**DOI:** 10.1371/journal.pone.0351405.r001

**Authors:** Michael Burger

6 Apr 2026

PONE-D-26-12461

Unimodal vs. multimodal deep learning for non-invasive MGMT promoter methylation prediction in glioblastoma: a systematic evaluation on the BraTS 2021 dataset

PLOS One

Dear Dr. Cadet,

Thank you for submitting your manuscript to PLOS ONE. After careful consideration, we feel that it has merit but does not fully meet PLOS ONE’s publication criteria as it currently stands. Therefore, we invite you to submit a revised version of the manuscript that addresses the points raised during the review process.

Please modify your manuscript according to the suggestions brought forward by the reviewers. Please discuss the reasons, where this might not be possible.

Please submit your revised manuscript by May 21 2026 11:59PM. If you will need more time than this to complete your revisions, please reply to this message or contact the journal office at plosone@plos.org. When you're ready to submit your revision, log on to https://www.editorialmanager.com/pone/ and select the 'Submissions Needing Revision' folder to locate your manuscript file.

Please include the following items when submitting your revised manuscript:

- A letter that responds to each point raised by the academic editor and reviewer(s). You should upload this letter as a separate file labeled 'Response to Reviewers'.
- A marked-up copy of your manuscript that highlights changes made to the original version. You should upload this as a separate file labeled 'Revised Manuscript with Track Changes'.
- An unmarked version of your revised paper without tracked changes. You should upload this as a separate file labeled 'Manuscript'.

If you would like to make changes to your financial disclosure, please include your updated statement in your cover letter. Guidelines for resubmitting your figure files are available below the reviewer comments at the end of this letter.

If applicable, we recommend that you deposit your laboratory protocols in protocols.io to enhance the reproducibility of your results. Protocols.io assigns your protocol its own identifier (DOI) so that it can be cited independently in the future. For instructions see: https://journals.plos.org/plosone/s/submission-guidelines#loc-laboratory-protocols. Additionally, PLOS ONE offers an option for publishing peer-reviewed Lab Protocol articles, which describe protocols hosted on protocols.io. Read more information on sharing protocols at https://plos.org/protocols?utm_medium=editorial-email&utm_source=authorletters&utm_campaign=protocols.

As the corresponding author, your ORCID iD is verified in the submission system and will appear in the published article. PLOS supports the use of ORCID, and we encourage all coauthors to register for an ORCID iD and use it as well. Please encourage your coauthors to verify their ORCID iD within the submission system before final acceptance, as unverified ORCID iDs will not appear in the published article. *Only* the individual author can complete the verification step; PLOS staff *cannot* verify ORCID iDs on behalf of authors.

We look forward to receiving your revised manuscript.

Kind regards,

Michael C Burger, M.D.

Academic Editor

PLOS One

**Journal Requirements:**

When submitting your revision, we need you to address these additional requirements.

1. Please ensure that your manuscript meets PLOS ONE's style requirements, including those for file naming. The PLOS ONE style templates can be found at

https://journals.plos.org/plosone/s/file?id=wjVg/PLOSOne_formatting_sample_main_body.pdf and

https://journals.plos.org/plosone/s/file?id=ba62/PLOSOne_formatting_sample_title_authors_affiliations.pdf

2. Please note that PLOS One has specific guidelines on code sharing for submissions in which author-generated code underpins the findings in the manuscript. In these cases, we expect all author-generated code to be made available without restrictions upon publication of the work. Please review our guidelines at https://journals.plos.org/plosone/s/materials-and-software-sharing#loc-sharing-code and ensure that your code is shared in a way that follows best practice and facilitates reproducibility and reuse.

3. Thank you for stating the following financial disclosure:

“FO is supported by a PhD grant from the Region Reunion and European Union (FEDER) under European Operational Program FEDER-FSE+ REUNION –2021/2027 file number 2021037, tiers 227180.”

Please state what role the funders took in the study.  If the funders had no role, please state: "The funders had no role in study design, data collection and analysis, decision to publish, or preparation of the manuscript."

If this statement is not correct you must amend it as needed.

Please include this amended Role of Funder statement in your cover letter; we will change the online submission form on your behalf.

4. PLOS requires an ORCID iD for the corresponding author in Editorial Manager on papers submitted after December 6th, 2016. Please ensure that you have an ORCID iD and that it is validated in Editorial Manager. To do this, go to ‘Update my Information’ (in the upper left-hand corner of the main menu), and click on the Fetch/Validate link next to the ORCID field. This will take you to the ORCID site and allow you to create a new iD or authenticate a pre-existing iD in Editorial Manager.

5. If the reviewer comments include a recommendation to cite specific previously published works, please review and evaluate these publications to determine whether they are relevant and should be cited. There is no requirement to cite these works unless the editor has indicated otherwise.

[Note: HTML markup is below. Please do not edit.]

Reviewers' comments:

Reviewer's Responses to Questions

**Comments to the Author**

1. Is the manuscript technically sound, and do the data support the conclusions?

The manuscript must describe a technically sound piece of scientific research with data that supports the conclusions. Experiments must have been conducted rigorously, with appropriate controls, replication, and sample sizes. The conclusions must be drawn appropriately based on the data presented.

Reviewer #1: Partly

Reviewer #2: No

**********

2. Has the statistical analysis been performed appropriately and rigorously?

Reviewer #1: N/A

Reviewer #2: No

**********

3. Have the authors made all data underlying the findings in their manuscript fully available?

The PLOS Data policy requires authors to make all data underlying the findings described in their manuscript fully available without restriction, with rare exception (please refer to the Data Availability Statement in the manuscript PDF file). The data should be provided as part of the manuscript or its supporting information, or deposited to a public repository. For example, in addition to summary statistics, the data points behind means, medians and variance measures should be available. If there are restrictions on publicly sharing data—e.g. participant privacy or use of data from a third party—those must be specified.

Reviewer #1: No

Reviewer #2: Yes

**********

4. Is the manuscript presented in an intelligible fashion and written in standard English?

PLOS ONE does not copyedit accepted manuscripts, so the language in submitted articles must be clear, correct, and unambiguous. Any typographical or grammatical errors should be corrected at revision, so please note any specific errors here.

Reviewer #1: Yes

Reviewer #2: Yes

**********

5. Review Comments to the Author

Please use the space provided to explain your answers to the questions above. You may also include additional comments for the author, including concerns about dual publication, research ethics, or publication ethics. (Please upload your review as an attachment if it exceeds 20,000 characters)

Reviewer #1: I congratulate you on the idea behind this work. I find the systematic and controlled evaluation of unimodality versus multimodality particularly useful, even in practical, real-world applications. However, this study appears very similar to the 2023 paper “MGMT promoter methylation status prediction using MRI scans? An extensive experimental evaluation of deep learning models.” Moreover, in its current form, the manuscript has several critical issues:

1) *Unclear and insufficiently justified slice selection*: The authors describe the number of slices used but do not specify clearly which slices from the volume are actually selected. This is a crucial point, especially with few slices: how do you ensure that at least one contains the tumor or an informative region? If the selected slices do not include the tumor, the prediction risks becoming essentially random or based on irrelevant signals. This aspect needs better clarification and discussion as a methodological limitation.

2) *Inaccuracies in the total number of experiments*: At line 313, the product 11×6×2×3 equals 396, but the text also mentions 369. Additionally, 1188 = 396×3 is reported, but the path to the total of 1584 experiments is not shown precisely and transparently.

3) *Inaccuracies in bibliographic references*: In Table 2, “Robinet et al.24” and “Saeed et al.25” appear, but in the bibliography, corresponds to Robinet et al. 2023 and to Saeed et al. 2023. The same error repeats in Table 3 with “Robinet et al.24” and “Saeed et al.25.”

4) *Overly strong phrasing in concluding statements*: The authors claim that (i) multimodality does not improve performance, (ii) the bottleneck is not the number of modalities but the quality/specificity of features, and (iii) T2w coronal should be prioritized for future data collection. These conclusions stem from a rather specific framework based on a single backbone family (VGG-16 from 2014) and relatively simple fusion strategies. The phrasing should be more cautious, e.g., “within the tested 2D VGG-16 framework…”

5) *Weak validation framework*: All experiments are evaluated on a single training/testing split, despite 5-fold cross-validation within training. Performances could thus be substantially influenced by the initial split's random seed. To demonstrate true robustness, a nested cross-validation or repetition over multiple splits would be preferable.

6) *Comparisons with state-of-the-art not always correct or fair*: The comparison tables mix studies on different datasets and test sets. As presented, this risks being incorrect or at least misleading. The limits of comparability need clearer explanation.

7) *Inconsistent terminology*: Terminology is not fully uniform in several places throughout the manuscript. A stylistic and terminological review would improve overall clarity.

8) *Inconsistency between Table 2 and text on the best model*: The text states that for the best model with transfer learning in the coronal plane, “the best model is obtained by using T2w images with only 1 slice,” with validation accuracy 0.6222 and test accuracy 0.5966. However, Table 2 shows the best TL model using 24 slices. It must be clarified which is actually the best model: 1 slice or 24 slices?

9) *Overly strong discussion of T2w coronal*: The observation is interesting, but the result remains highly dataset-specific and architecture-specific. Thus, the recommendation to prioritize T2w coronal in future data collection should be expressed more cautiously.”

Reviewer #2: This manuscript presents a systematic evaluation of unimodal versus multimodal deep learning approaches for non-invasive prediction of MGMT promoter methylation status in glioblastoma using the BraTS 2021 dataset.

While the topic is of high clinical relevance and the attempt to comprehensively benchmark multiple configurations is commendable, the study suffers from substantial methodological, analytical, and interpretative limitations that significantly weaken the validity of its conclusions.

First, the overall scientific objective remains insufficiently defined and internally inconsistent. The authors state that their primary aim is not to propose a new architecture but rather to characterize optimal input configurations (lines 113–116). However, they simultaneously derive strong clinical recommendations, such as prioritizing T2-weighted coronal acquisitions (lines 42–44, 499–500). This discrepancy between an exploratory methodological study and deterministic clinical conclusions is problematic and suggests an overextension of the presented results.

My major concern relates to the study design and data handling. The dataset is split into an 80/20 train-test partition prior to applying five-fold cross-validation (lines 123–124, 185–186). This approach is suboptimal and introduces a risk of bias, as it does not constitute a truly independent test evaluation and may lead to implicit information leakage or indirect model tuning on the test set. A nested cross-validation scheme or an external validation cohort would have been more appropriate, particularly given the limited dataset size. Moreover, the absence of any external validation (lines 471–474) severely limits the generalizability of the findings and precludes any meaningful clinical interpretation.

The methodological framework itself raises further concerns. The use of 2D slice-based analysis instead of volumetric 3D modeling (lines 138–142, 463–466) represents a significant limitation, as it disregards essential spatial information inherent to MRI data. This is particularly critical in glioblastoma, where tumor heterogeneity and infiltration patterns are inherently three-dimensional. Additionally, the study does not incorporate tumor segmentation, instead relying on whole-brain images (lines 456–462). This decision substantially reduces the signal-to-noise ratio and likely dilutes any biologically relevant features associated with MGMT status. While the authors acknowledge this as a limitation, it should be considered a fundamental methodological flaw rather than a secondary issue.

The choice of model architecture is also insufficiently justified. The exclusive use of VGG-16 (lines 107–108, 202–206), a relatively outdated architecture in the context of modern medical imaging, is not adequately motivated, and no comparison with more advanced models such as 3D CNNs, EfficientNet variants, or transformer-based approaches is provided. This limits the study’s relevance to the current state of the field.

From a statistical perspective, the analysis is notably incomplete. The authors report average performance metrics across cross-validation folds (lines 236–237, 302–303) but do not provide measures of variability such as standard deviations or confidence intervals. Furthermore, no statistical testing is performed, despite claims that no meaningful differences exist between fusion strategies (line 405). Such statements are therefore not substantiated. The evaluation is restricted to accuracy and AUC (lines 189–190), without consideration of additional clinically relevant metrics such as calibration, precision-recall analysis, or decision curve analysis. Given the modest performance levels reported, a more comprehensive evaluation framework would have been essential.

The interpretation of results is another major weakness of the manuscript. The authors attribute the observed performance ceiling primarily to dataset-related factors such as label noise and heterogeneity (lines 361–368, 42–43), yet alternative explanations—most notably the limitations of the chosen methodology—are not sufficiently explored. The claim that MGMT prediction is “fundamentally constrained” by dataset properties is therefore speculative and not convincingly supported by the presented data. Similarly, the recommendation to prioritize T2-weighted coronal imaging (lines 42–44, 499–500) is not justified, as the reported performance differences are marginal, lack statistical validation, and may reflect dataset-specific biases rather than true biological relevance.

Several inconsistencies and formal issues further detract from the manuscript’s rigor. The reported patient numbers are not consistently described (lines 32 vs. 121–122), and there are minor but noticeable errors, including inconsistent reporting of model counts (line 313) and typographical inaccuracies (e.g., “trained without RL” instead of TL, line 318). While individually minor, these issues contribute to an overall impression of insufficient methodological precision.

Despite these limitations, the study does have strengths. The large number of evaluated configurations (lines 33–34, 315) and the systematic comparison of multimodal fusion strategies represent valuable contributions. The authors also provide a generally transparent discussion of the performance limitations and acknowledge key methodological shortcomings.

In conclusion, this manuscript can be interpreted as a negative benchmark study demonstrating the limited performance of current deep learning approaches for MGMT prediction on the BraTS 2021 dataset. However, due to significant methodological weaknesses, insufficient statistical analysis, and overinterpretation of results, the current version does not support the strength of its conclusions. Substantial revisions would be required, including improved experimental design, incorporation of tumor-focused modeling approaches, more rigorous statistical evaluation, and a more cautious interpretation of findings.

In its present form, the manuscript is not suitable for publication, but it could provide a useful contribution after major revision.

**********

6. PLOS authors have the option to publish the peer review history of their article (what does this mean?). If published, this will include your full peer review and any attached files.

If you choose “no”, your identity will remain anonymous but your review may still be made public.

**Do you want your identity to be public for this peer review?** For information about this choice, including consent withdrawal, please see our Privacy Policy.

Reviewer #1: No

Reviewer #2: No

**********

[NOTE: If reviewer comments were submitted as an attachment file, they will be attached to this email and accessible via the submission site. Please log into your account, locate the manuscript record, and check for the action link "View Attachments". If this link does not appear, there are no attachment files.]

To ensure your figures meet our technical requirements, please review our figure guidelines: https://journals.plos.org/plosone/s/figures

You may also use PLOS’s free figure tool, NAAS, to help you prepare publication quality figures: https://journals.plos.org/plosone/s/figures#loc-tools-for-figure-preparation.

NAAS will assess whether your figures meet our technical requirements by comparing each figure against our figure specifications.

# Author comment: Author response to Decision Letter 1

**DOI:** 10.1371/journal.pone.0351405.r002

7 May 2026

Response to Reviewers

Unimodal vs. multimodal deep learning for non-invasive MGMT promoter methylation prediction in glioblastoma: a systematic evaluation on the BraTS 2021 dataset

Submitted to PLOS ONE

Response to Reviewer #1

We thank Reviewer #1 for the positive assessment of the study's systematic design and for the detailed, constructive comments. We address each point below.

Comment 1 — Slice selection methodology

Reviewer comment:

Unclear and insufficiently justified slice selection. The authors describe the number of slices used but do not specify clearly which slices from the volume are actually selected. This is a crucial point, especially with few slices: how do you ensure that at least one contains the tumor or an informative region? If the selected slices do not include the tumor, the prediction risks becoming essentially random or based on irrelevant signals.

Response:

We thank the reviewer for raising this important methodological point. The slice selection strategy is described in the Methods section (Pre-processing subsection): slices are selected starting from the middle of the volume and expanding symmetrically outward. For example, with n=1 slice, the central slice is selected; with n=3, the central slice and one on each side; and so on. This center-out strategy is motivated by the fact that, in the BraTS 2021 dataset, MRI scans are skull-stripped and co-registered to a standard template, making the tumor region statistically likely to be near the center of the volume.

We fully acknowledge that this heuristic does not guarantee tumor coverage for all patients, particularly for small or peripherally located tumors, and that this constitutes a genuine methodological limitation. We have added a dedicated clarification in the Pre-processing subsection and strengthened the corresponding limitation discussion in the Study Limitations section. We note that this limitation is, in fact, consistent with the whole-brain, segmentation-free paradigm of this study, which deliberately avoids the use of tumor masks in order to evaluate the raw discriminative capacity of mpMRI sequences — a choice that is explicitly acknowledged as a limitation (see Absence of tumor segmentation, Study Limitations section).

Manuscript changes:

Pre-processing subsection: Added explicit description of the center-out slice selection strategy with the rationale based on co-registration to standard template. (l.175-178): “For this study, the standardization is used with its parameters computed for each scan. In the final pre-processing step, a defined number of slices is extracted symmetrically around the central slice of the scan. When a single slice is selected, only the central one is retained; when multiple slices are chosen, the selection extends outward from the center in both directions.”

Study Limitations section: The existing paragraph on 2D slice-based architecture is corrected to address slice selection uncertainty as a specific sub-limitation (l.492-493): “A major limitation of this study is the absence of tumor region segmentation, both in slice selection and at the model input level.”

Comment 2 — Arithmetic errors in experiment count

Reviewer comment:

Inaccuracies in the total number of experiments. At line 313, the product 11×6×2×3 equals 396, but the text also mentions 369. Additionally, 1188 = 396×3 is reported, but the path to the total of 1,584 experiments is not shown precisely and transparently.

Response:

The reviewer is correct. There are two distinct errors in the manuscript that we correct as follows:

• Error 1: "369" in the text is a typographical error. The correct product 11 × 6 × 2 × 3 = 396, and this is the correct value to report.

• Error 2: The figure of 1,584 total experiments cited in the Abstract corresponds to the sum of all unimodal and multimodal experiments but is an error: 396 (unimodal: 4 sequences × 8 slice counts × 2 TL conditions × 3 planes = 192) and 1,188 (multimodal with 3 fusion strategies: 396 × 3). The correct breakdown is: 192 (unimodal) + 1,188 (multimodal) = 1,380. This decomposition was not made explicit in the text, and we correct this.

We acknowledge that the figure of 1,584 given in the Abstract requires correction and precise justification, which we provide in the revised manuscript.

Manuscript changes:

Line 323: '369' corrected to '396'.

Abstract: The total experiment count is corrected, and the full arithmetic decomposition (unimodal + multimodal) is made explicit with a parenthetical breakdown (l.34): “exploring 1,380 experimental configurations (unimodal: 192; multimodal: 1,188).”

Comment 3 — Reference numbering errors in Tables 2 and 3

Reviewer comment:

Inaccuracies in bibliographic references. In Table 2, "Robinet et al.24" and "Saeed et al.25" appear, but in the bibliography, [24] corresponds to Robinet et al. 2023 and [25] to Saeed et al. 2023. The same error repeats in Table 3.

Response:

The reviewer is correct. The reference numbers in Tables 2 and 3 are misaligned with the bibliography. Upon verification, the correct reference numbers are [19] for Robinet et al. 2023 and [20] for Saeed et al. 2023 in the current bibliography numbering. We correct these reference numbers in both tables.

Manuscript changes:

Tables 2 and 3: Reference corrected from [24] to [19] for Robinet et al. and from [25] to [20] for Saeed et al. The full bibliography is verified for internal consistency.

Comment 4 — Overconfident concluding statements

Reviewer comment:

Overly strong phrasing in concluding statements. The authors claim that (i) multimodality does not improve performance, (ii) the bottleneck is not the number of modalities but the quality/specificity of features, and (iii) T2w coronal should be prioritized for future data collection. These conclusions stem from a rather specific framework based on a single backbone family (VGG-16 from 2014) and relatively simple fusion strategies. The phrasing should be more cautious.

Response:

We agree with the reviewer that these conclusions require more careful scoping. The study is explicitly designed as a systematic benchmark within a 2D VGG-16 framework, and the generalizability of these findings to other architectures such as 3D approaches, or more sophisticated fusion strategies cannot be claimed from these results alone. We have revised the relevant passages throughout the manuscript — in the Abstract, Discussion, and Conclusion — to systematically scope all three claims with the qualifier "within the tested 2D VGG-16 framework" or equivalent.

We note, however, that our finding regarding the multimodal performance ceiling is consistent with results reported by Saeed et al. (2023) using a broader set of architectures including ResNet-34 and EfficientNet-B1, suggesting that the limitation may extend beyond VGG-16 in this dataset. This convergent evidence is retained in the revised Discussion.

Manuscript changes:

Abstract (l.42): “These findings suggest, for the tested framework in this study, that MGMT…”

Discussion (l.405-407): “A central and robust finding of this study is that combining multiple MRI sequences through any of the three fusion strategies — early, intermediate, or late — does not yield better-performing models than the best unimodal approach within the tested 2D VGG-16 framework.”

Discussion (l.413-416): “The behavior observed in our model suggests that, at least in the present experimental setting, performance bottleneck appear to stem primarily from insufficient feature specificity and quality, particularly the absence of explicit tumor region localization, rather than from a lack of input modality diversity.”

Conclusion (l.554-555): “Based on our empirical findings, T2w coronal images appear to be more suited to develop a better model, …”

Comment 5 — Single train/test split validation

Reviewer comment:

Weak validation framework. All experiments are evaluated on a single training/testing split, despite 5-fold cross-validation within training. Performances could thus be substantially influenced by the initial split's random seed. To demonstrate true robustness, a nested cross-validation or repetition over multiple splits would be preferable.

Response:

We acknowledge this limitation. The use of a single 80/20 train-test split does introduce sensitivity to the random seed of that partition, and nested cross-validation would in principle provide a more robust variance estimate. We fully recognize this as a methodological weakness.

However, we respectfully note that repeating all experiments over multiple splits is computationally prohibitive given the scale of this study: 1,380 trained models already represent a substantial computational investment because it took approximately 1 month of computing to obtain the results in this study despite optimization and parallelization. Multiplying this by N independent splits (e.g., N=5 for a nested scheme) would require a fivefold increase in compute. This constraint is acknowledged transparently in the Study Limitations section. We note furthermore that the single-split design is consistent with the majority of comparable published benchmarks on this dataset (including the BraTS 2021 challenge itself, Kim et al. 2023, and Robinet et al. 2023), making our results at least comparably constrained.

We have strengthened the limitations section to make this constraint explicit, noted the risk of split-dependent variance, and added the random seed of the split to the Methods section to ensure reproducibility.

Manuscript changes:

Methods (l.126-127): “with a ratio 8:2 with respect to the initial distribution with a random seed for reproducibility.”

Study Limitations section: New paragraph explicitly addressing the single-split design, the associated risk of seed-dependent variance, and the computational rationale for the chosen design (l.514-519): “Single split design. The dataset was partitioned into training and test sets using a fixed random seed to ensure reproducibility. While this approach guarantees consistent splits across runs, it introduces a risk of seed-dependent variance, which represents a less robust alternative to methodologies such as nested cross-validation. However, the current pipeline already operates near its computational limits despite parallelization and runtime optimizations. Expanded computational capacity would be a prerequisite for moving toward a more reliable evaluation strategy.”

Comment 6 — Comparability of state-of-the-art tables

Reviewer comment:

Comparisons with state-of-the-art not always correct or fair. The comparison tables mix studies on different datasets and test sets. As presented, this risks being incorrect or at least misleading. The limits of comparability need clearer explanation.

Response:

The reviewer raises a valid and important point. The limitations of these comparisons are already partially acknowledged in the tables footnotes ("* indicates that the results are difficult to compare"). The paragraph before the Table 2 is revised and a paragraph before Table 3 is added to systematically enumerates the sources of non-comparability: different datasets (BraTS 2021 public vs. private institutional cohorts) and different evaluation protocols (test set vs. cross-validation only). The term ‘comparison’ is replaced by ‘contextualization against literature’ in the text to better reflect the intended purpose of these tables.

Manuscript changes:

Table 2: The explanation is already in the footnote in the table and in the paragraph before the table (l.300-303): “Table 2 allows a contextualization of our best results obtained with the unimodal approach against the literature. The test set used by the competition winner is not available to us. The study by Robinet et al. uses a private dataset, and Saeed et al. does not have a test set since they use cross-validation.”

Table 3: The footnote is already present in the initial manuscript, and a paragraph is added in the revised manuscript (l.351-353): “Results in Table 3 are accompanied by multimodal results found in the state-of-the-art. In the study of Robinet et al., they use a private test set not accessible for us. Saeed et al. uses cross-validation to train their model; therefore, they do not have any results on a test set.”

Comment 7 — Terminological inconsistencies

Reviewer comment:

Inconsistent terminology. Terminology is not fully uniform in several places throughout the manuscript. A stylistic and terminological review would improve overall clarity.

Response:

We thank the reviewer for this observation. A full terminological audit of the manuscript has been conducted. Key inconsistencies identified and corrected include:

(1) 'without RL' corrected to 'without TL' (line 328, noted also by Reviewer #2);

(2) inconsistent capitalization of 'Coronal', 'Sagittal', and 'Axial' when used as plane descriptors (standardized);

Manuscript changes:

Full manuscript: Terminological standardization as described above. Line 328: 'RL' corrected to 'TL'.

Comment 8 — Inconsistency between Table 2 and text (1 slice vs. 24 slices)

Reviewer comment:

Inconsistency between Table 2 and text on the best model. The text states that for the best model with transfer learning in the coronal plane, 'the best model is obtained by using T2w images with only 1 slice,' with validation accuracy 0.6222 and test accuracy 0.5966. However, Table 2 shows the best TL model using 24 slices.

Response:

The reviewer is correct. There is an inconsistency between the body text and Table 2. The text correctly describes the best TL model with T2w coronal as the 1-slice configuration (validation accuracy 0.6222, test accuracy 0.5966).

The 2nd line in Table 2 mistakenly showed the model with the best performance on the test set. The row is corrected to display the model with the best performance on validation set and with TL.

Manuscript changes:

Table 2, row 'VGG-16 This study (TL = Yes)': Results with the correct model is shown.

Comment 9 — Overconfident recommendation on T2w coronal

Reviewer comment:

Overly strong discussion of T2w coronal. The observation is interesting, but the result remains highly dataset-specific and architecture-specific. The recommendation to prioritize T2w coronal in future data collection should be expressed more cautiously.

Response:

We fully agree. This point is closely related to Comment #4, and the same revision applies. The recommendation regarding T2w coronal acquisitions is now explicitly qualified as a dataset-specific and framework-specific empirical observation, contingent on the 2D VGG-16 architecture and the BraTS 2021 dataset characteristics. The revised phrasing reads: 'Within the tested 2D VGG-16 framework on the BraTS 2021 dataset, T2w coronal images were consistently associated with the best-performing configurations. This observation may reflect dataset-specific imaging properties and should not be generalized without validation on independent cohorts and architectures.'

Manuscript changes:

Abstract (l.44-45): “… T2w coronal acquisitions could be more interesting in future data collection efforts.”

Discussion (l.390-393): “'Within the tested 2D VGG-16 framework on the BraTS 2021 dataset, T2w coronal images were consistently associated with the best-performing configurations. This finding may reflect dataset-specific imaging properties and should not be generalized without validation on independent cohorts and architectures.”

Conclusion (l.554-555): “Based on our empirical findings, T2w coronal images appear to be more suited to develop a better model, …”

Response to Reviewer #2

We thank Reviewer #2 for the thorough and rigorous evaluation. The reviewer correctly identifies several methodological limitations, and we respond to each point below with a combination of manuscript revisions and, where appropriate, scientific justification of the study design choices.

Comment 1 — Scientific objective and clinical overreach

Reviewer comment:

The overall scientific objective remains insufficiently defined and internally inconsistent. The authors state that their primary aim is not to propose a new architecture but rather to characterize optimal input configurations (lines 113-116). However, they simultaneously derive strong clinical recommendations, such

Attachment Submitted filename: Response_to_Reviewers_MGMT_PLOSONE_v2.docx

# Peer review: Decision Letter 1

**DOI:** 10.1371/journal.pone.0351405.r003

**Authors:** Michael Burger

20 May 2026

PONE-D-26-12461R1

Unimodal vs. multimodal deep learning for non-invasive MGMT promoter methylation prediction in glioblastoma: a systematic evaluation on the BraTS 2021 dataset

PLOS One

Dear Dr. Cadet,

Thank you for submitting your manuscript to PLOS ONE. After careful consideration, we feel that it has merit but does not fully meet PLOS ONE’s publication criteria as it currently stands. Therefore, we invite you to submit a revised version of the manuscript that addresses the points raised during the review process.

Please submit your revised manuscript by Jul 04 2026 11:59PM. If you will need more time than this to complete your revisions, please reply to this message or contact the journal office at plosone@plos.org. When you're ready to submit your revision, log on to https://www.editorialmanager.com/pone/ and select the 'Submissions Needing Revision' folder to locate your manuscript file.

Please include the following items when submitting your revised manuscript:

- A letter that responds to each point raised by the academic editor and reviewer(s). You should upload this letter as a separate file labeled 'Response to Reviewers'.
- A marked-up copy of your manuscript that highlights changes made to the original version. You should upload this as a separate file labeled 'Revised Manuscript with Track Changes'.
- An unmarked version of your revised paper without tracked changes. You should upload this as a separate file labeled 'Manuscript'.

If you would like to make changes to your financial disclosure, please include your updated statement in your cover letter. Guidelines for resubmitting your figure files are available below the reviewer comments at the end of this letter.

If applicable, we recommend that you deposit your laboratory protocols in protocols.io to enhance the reproducibility of your results. Protocols.io assigns your protocol its own identifier (DOI) so that it can be cited independently in the future. For instructions see: https://journals.plos.org/plosone/s/submission-guidelines#loc-laboratory-protocols. Additionally, PLOS ONE offers an option for publishing peer-reviewed Lab Protocol articles, which describe protocols hosted on protocols.io. Read more information on sharing protocols at https://plos.org/protocols?utm_medium=editorial-email&utm_source=authorletters&utm_campaign=protocols.

As the corresponding author, your ORCID iD is verified in the submission system and will appear in the published article. PLOS supports the use of ORCID, and we encourage all coauthors to register for an ORCID iD and use it as well. Please encourage your coauthors to verify their ORCID iD within the submission system before final acceptance, as unverified ORCID iDs will not appear in the published article. *Only* the individual author can complete the verification step; PLOS staff *cannot* verify ORCID iDs on behalf of authors.

We look forward to receiving your revised manuscript.

Kind regards,

Michael C Burger, M.D.

Academic Editor

PLOS One

Journal Requirements:

If the reviewer comments include a recommendation to cite specific previously published works, please review and evaluate these publications to determine whether they are relevant and should be cited. There is no requirement to cite these works unless the editor has indicated otherwise.

Please review your reference list to ensure that it is complete and correct. If you have cited papers that have been retracted, please include the rationale for doing so in the manuscript text, or remove these references and replace them with relevant current references. Any changes to the reference list should be mentioned in the rebuttal letter that accompanies your revised manuscript. If you need to cite a retracted article, indicate the article’s retracted status in the References list and also include a citation and full reference for the retraction notice.

Additional Editor Comments:

Please carefully check your manuscript, and resolve the inconsistencies still present, as summarized by the Reviewer.

[Note: HTML markup is below. Please do not edit.]

Reviewers' comments:

Reviewer's Responses to Questions

**Comments to the Author**

1. If the authors have adequately addressed your comments raised in a previous round of review and you feel that this manuscript is now acceptable for publication, you may indicate that here to bypass the “Comments to the Author” section, enter your conflict of interest statement in the “Confidential to Editor” section, and submit your "Accept" recommendation.

Reviewer #2: (No Response)

**********

2. Is the manuscript technically sound, and do the data support the conclusions?

The manuscript must describe a technically sound piece of scientific research with data that supports the conclusions. Experiments must have been conducted rigorously, with appropriate controls, replication, and sample sizes. The conclusions must be drawn appropriately based on the data presented.

Reviewer #2: (No Response)

**********

3. Has the statistical analysis been performed appropriately and rigorously?

Reviewer #2: (No Response)

**********

4. Have the authors made all data underlying the findings in their manuscript fully available?

The PLOS Data policy requires authors to make all data underlying the findings described in their manuscript fully available without restriction, with rare exception (please refer to the Data Availability Statement in the manuscript PDF file). The data should be provided as part of the manuscript or its supporting information, or deposited to a public repository. For example, in addition to summary statistics, the data points behind means, medians and variance measures should be available. If there are restrictions on publicly sharing data—e.g. participant privacy or use of data from a third party—those must be specified.

Reviewer #2: (No Response)

**********

5. Is the manuscript presented in an intelligible fashion and written in standard English?

PLOS ONE does not copyedit accepted manuscripts, so the language in submitted articles must be clear, correct, and unambiguous. Any typographical or grammatical errors should be corrected at revision, so please note any specific errors here.

Reviewer #2: (No Response)

**********

6. Review Comments to the Author

Please use the space provided to explain your answers to the questions above. You may also include additional comments for the author, including concerns about dual publication, research ethics, or publication ethics. (Please upload your review as an attachment if it exceeds 20,000 characters)

Reviewer #2: The revised manuscript has improved substantially compared with the original submission. The authors have addressed the majority of the major methodological and interpretative concerns in a thoughtful and scientifically appropriate manner. In particular, the manuscript is now more consistently framed as an exploratory benchmark study rather than a clinically actionable predictive model, which significantly strengthens the overall scientific positioning of the work.

The revised discussion of the study limitations is considerably more balanced and transparent. The authors now appropriately acknowledge the potential influence of methodological limitations such as the absence of tumor segmentation, the use of a 2D framework, the single-split validation design, and the restriction to a single architectural backbone. The clarification regarding the absence of test-set leakage is also satisfactory. Furthermore, the addition of variability measures, more cautious interpretation of multimodal fusion results, and expanded discussion of alternative explanations for the observed performance ceiling all improve the rigor and credibility of the manuscript.

Importantly, the authors provide a convincing justification for the use of a fixed VGG-16 backbone within the context of a controlled combinatorial benchmark study. While the framework remains methodologically limited compared with current state-of-the-art radiogenomic approaches, the manuscript now clearly acknowledges these limitations and appropriately scopes its conclusions.

However, after reviewing both the rebuttal letter and the revised manuscript, there appear to be several inconsistencies between the modifications described in the response and the actual text currently present in the manuscript. Some statements that were reportedly revised remain unchanged or insufficiently softened in the uploaded version. For example, the Abstract still contains relatively strong wording such as “fundamentally constrained” and “should be prioritized in future data collection efforts,” despite the authors indicating that these conclusions had been reformulated more cautiously. Similarly, the total number of experimental configurations in the Abstract still appears inconsistent with the corrected arithmetic described in the rebuttal letter. In addition, some phrasing in the Introduction continues to imply stronger clinical applicability than intended according to the revised study framing.

These issues are relatively minor and do not require additional experimental work, but they should be corrected to ensure consistency between the rebuttal and the revised manuscript itself. I therefore recommend a final minor revision focused on editorial consistency and precise alignment of the manuscript text with the authors’ stated revisions.

Provided these remaining textual inconsistencies are corrected, I believe the manuscript would be acceptable for publication in its intended journal context.

**********

7. PLOS authors have the option to publish the peer review history of their article (what does this mean?). If published, this will include your full peer review and any attached files.

If you choose “no”, your identity will remain anonymous but your review may still be made public.

**Do you want your identity to be public for this peer review?** For information about this choice, including consent withdrawal, please see our Privacy Policy.

Reviewer #2: No

**********

[NOTE: If reviewer comments were submitted as an attachment file, they will be attached to this email and accessible via the submission site. Please log into your account, locate the manuscript record, and check for the action link "View Attachments". If this link does not appear, there are no attachment files.]

To ensure your figures meet our technical requirements, please review our figure guidelines: https://journals.plos.org/plosone/s/figures

You may also use PLOS’s free figure tool, NAAS, to help you prepare publication quality figures: https://journals.plos.org/plosone/s/figures#loc-tools-for-figure-preparation.

NAAS will assess whether your figures meet our technical requirements by comparing each figure against our figure specifications.

# Author comment: Author response to Decision Letter 2

**DOI:** 10.1371/journal.pone.0351405.r004

26 May 2026

Response to Reviewer — Minor Revision

Unimodal vs. multimodal deep learning for non-invasive MGMT promoter methylation prediction in glioblastoma: a systematic evaluation on the BraTS 2021 dataset

Submitted to PLOS ONE

Response to Reviewer #2

We thank Reviewer #2 for the thorough and constructive re-reading of the revised manuscript. We note with appreciation that the reviewer considers the work substantially improved and acceptable for publication after minor editorial corrections. We address the four identified inconsistencies below.

General remark. The reviewer correctly observes that several formulations described as revised in the rebuttal letter were not consistently implemented in the uploaded manuscript. We have now verified the full manuscript against every stated commitment in the response letter and corrected all outstanding discrepancies. The five specific issues are detailed below.

Inconsistency 1 — Abstract — “Fundamentally constrained”

Reviewer observation:

The Abstract still contains relatively strong wording such as “fundamentally constrained” [...] despite the authors indicating that these conclusions had been reformulated more cautiously.

Analysis:

In the rebuttal letter (Response to Reviewer #2, Comment 7), we committed to presenting the performance ceiling as a co-hypothesis between dataset-related and methodology-related factors. The term “fundamentally constrained” overstates the certainty of our interpretation.

The Discussion section was correctly revised to reflect this nuance (co-hypothesis framing, Kim et al. external evidence). However, the Abstract retained the original phrasing. The addition of the qualifier “, for the tested framework in this study,” introduced in the revised manuscript partially addresses the scoping concern but does not resolve the overstatement carried by “fundamentally constrained”. We therefore revise this phrase in the Abstract to achieve full consistency with the Discussion.

The term of “fundamentally constrained” is replaced with “appears primarily limited” to better align the Abstract with both the Discussion and the Introduction. The qualifier “appears primarily” acknowledges that methodological factors may also contribute, which is consistent with our stated position in the rebuttal.

Manuscript change:

Abstract (l.43): “fundamentally constrained” → “appears primarily limited”.

Inconsistency 2 — Abstract — T2w coronal phrasing

Reviewer observation:

The Abstract still contains relatively strong wording such as [...] “should be prioritized in future data collection efforts” despite the authors indicating that these conclusions had been reformulated more cautiously.

Analysis:

The rebuttal letter committed to replacing the prescriptive phrasing “should be prioritized” with a dataset-specific empirical observation. The revised manuscript introduced “could be more interesting” (Abstract) and “T2w coronal images appear to be more suited to develop a better model” (Conclusion). While these represent an improvement over the original, the Abstract phrasing “could be more interesting” remains imprecise and does not adequately convey the conditionality and dataset-specificity of the observation. We replace it with a formulation that is explicitly scoped and empirically grounded, consistent with the Discussion wording already in the revised manuscript.

Manuscript changes:

Abstract (l.44-45): “and that T2w coronal acquisitions could be more interesting in future data collection efforts” → “In this setting, T2w coronal images were associated with the most competitive configurations and may warrant further investigation in future studies”.

Inconsistency 3 — Introduction — Experiment count inconsistency (“over 1,300” vs. 1,380)

Reviewer observation:

The total number of experimental configurations in the Abstract still appears inconsistent with the corrected arithmetic described in the rebuttal letter.

Analysis:

The rebuttal letter committed to correcting the total experiment count and providing the explicit breakdown 192 (unimodal) + 1,188 (multimodal) = 1,380. The Abstract was correctly updated to “1,380 experimental configurations (unimodal: 192; multimodal: 1,188)”. However, the Introduction paragraph (objective statement) still reads “over 1,300 model configurations,” which is inconsistent with 1,380 and contradicts the explicit corrected figure. We replace this with the exact figure.

Manuscript change:

Introduction (l.108-109): “we trained and evaluated over 1,300 model configurations” → “we trained and evaluated 1,380 model configurations (192 unimodal; 1,188 multimodal)”.

Inconsistency 4 — Introduction — Residual clinical tone (“clinically critical task”)

Reviewer observation:

Some phrasing in the Introduction continues to imply stronger clinical applicability than intended according to the revised study framing.

Analysis:

The rebuttal letter (Response to Reviewer #2, Comment 1) committed to reframing the study consistently as an exploratory benchmark and removing prescriptive clinical language from the Introduction objective statement. The revised manuscript introduced the explicit scope qualifier “within the tested 2D VGG-16 framework” and replaced “should be prioritized” with softer formulations. However, the concluding clause of the objective statement in the Introduction (“thereby offering preliminary insights that could be more efficient for future data collection and model development in this clinically critical task”) retains a prescriptive tone inconsistent with the exploratory benchmark framing. Specifically, “clinically critical task” implies the models could be clinically deployed, which is explicitly contradicted by the Study Limitations and Discussion sections. We revise this clause to neutral, methodologically scoped language.

Manuscript change:

Introduction (l.116-118): “… thereby offering preliminary insights that could be more efficient for future data collection and model development in this clinically critical task.” → “Accordingly, the present study should be interpreted as an exploratory benchmark intended to inform subsequent methodological work and future data collection strategies”.

Inconsistency 5 — Results — Residual internal inconsistency

Analysis:

During the final consistency check, we identified and corrected one remaining internal inconsistency in the manuscript itself: the caption associated with the best coronal T2w configuration in Figure 5 still referred to 40 slices, whereas the corrected body text indicated 32 slices. This discrepancy has now been corrected.

Manuscript change:

Results (l.275): “… T2w modality and 40 slices …” → “… T2w modality and 32 slices …”.

Attachment Submitted filename: 23052026_Response_to_Reviewers_v2.docx

# Editor report: Decision Letter 2

**DOI:** 10.1371/journal.pone.0351405.r005

**Authors:** Michael Burger

27 May 2026

Unimodal vs. multimodal deep learning for non-invasive MGMT promoter methylation prediction in glioblastoma: a systematic evaluation on the BraTS 2021 dataset

PONE-D-26-12461R2

Dear Dr. Cadet,

We’re pleased to inform you that your manuscript has been judged scientifically suitable for publication and will be formally accepted for publication once it meets all outstanding technical requirements.

Within one week, you’ll receive an e-mail detailing the required amendments. When these have been addressed, you’ll receive a formal acceptance letter and your manuscript will be scheduled for publication.

An invoice will be generated when your article is formally accepted. Please note, if your institution has a publishing partnership with PLOS and your article meets the relevant criteria, all or part of your publication costs will be covered. Please make sure your user information is up-to-date by logging into Editorial Manager at Editorial Manager® and clicking the ‘Update My Information' link at the top of the page. For questions related to billing, please contact billing support.

If your institution or institutions have a press office, please notify them about your upcoming paper to help maximize its impact. If they’ll be preparing press materials, please inform our press team as soon as possible -- no later than 48 hours after receiving the formal acceptance. Your manuscript will remain under strict press embargo until 2 pm Eastern Time on the date of publication. For more information, please contact onepress@plos.org.

Kind regards,

Michael C Burger, M.D.

Academic Editor

PLOS One

Additional Editor Comments (optional):

Reviewers' comments:

# Editor report: Acceptance letter

**DOI:** 10.1371/journal.pone.0351405.r006

**Authors:** Michael Burger

PONE-D-26-12461R2

PLOS One

Dear Dr. Cadet,

I'm pleased to inform you that your manuscript has been deemed suitable for publication in PLOS One. Congratulations! Your manuscript is now being handed over to our production team.

At this stage, our production department will prepare your paper for publication. This includes ensuring the following:

* All references, tables, and figures are properly cited

* All relevant supporting information is included in the manuscript submission,

* There are no issues that prevent the paper from being properly typeset

You will receive further instructions from the production team, including instructions on how to review your proof when it is ready. Please keep in mind that we are working through a large volume of accepted articles, so please give us a few days to review your paper and let you know the next and final steps.

Lastly, if your institution or institutions have a press office, please let them know about your upcoming paper now to help maximize its impact. If they'll be preparing press materials, please inform our press team within the next 48 hours. Your manuscript will remain under strict press embargo until 2 pm Eastern Time on the date of publication. For more information, please contact onepress@plos.org.

You will receive an invoice from PLOS for your publication fee after your manuscript has reached the completed accept phase. If you receive an email requesting payment before acceptance or for any other service, this may be a phishing scheme. Learn how to identify phishing emails and protect your accounts at https://explore.plos.org/phishing.

If we can help with anything else, please email us at customercare@plos.org.

Thank you for submitting your work to PLOS ONE and supporting open access.

Kind regards,

PLOS ONE Editorial Office Staff

on behalf of

Dr. Michael C Burger

Academic Editor

PLOS One
